# Body mass index stratified meta-analysis of genome-wide association studies of polycystic ovary syndrome in women of European ancestry

**DOI:** 10.1186/s12864-024-09990-w

**Published:** 2024-02-26

**Authors:** Kharis Burns, Benjamin H. Mullin, Loes M. E. Moolhuijsen, Triin Laisk, Jaakko S. Tyrmi, Jinrui Cui, Ky’Era V. Actkins, Yvonne V. Louwers, Estonian Biobank Research Team, Andres Metspalu, Lili Milani, Tõnu Esko, Mari Nelis, Georgi Hudjashov, Lea K. Davis, Frank Dudbridge, Ricardo Azziz, Mark O. Goodarzi, Hannele Laivuori, Reedik Mägi, Jenny A. Visser, Joop S. E. Laven, Scott G. Wilson, FinnGen, International PCOS Consortium, Tugce Karaderi, Michelle R. Jones, Cindy Meun, Chunyan He, Alex Drong, Peter Kraft, Nan Lin, Hongyan Huang, Linda Broer, Richa Saxena, Andres Metspalu, Lili Milani, Tõnu Esko, Mari Nelis, Georgi Hudjashov, Margrit Urbanek, M. Geoffrey Hayes, Gudmar Thorleifsson, Juan Fernandez-Tajes, Anubha Mahajan, Timothy D. Spector, Barbara Obermayer-Pietsch, André G. Uitterlinden, Verneri Anttila, Benjamin M. Neale, Marjo-Riitta Jarvelin, Mark Daly, Bart Fauser, Irina Kowalska, Marianne Andersen, Ken Ong, Elisabet Stener-Victorin, David Ehrmann, Richard S. Legro, Andres Salumets, Mark I. McCarthy, Laure Morin-Papunen, Unnur Thorsteinsdottir, Kari Stefansson, Felix R. Day, Bronwyn G. A. Stuckey

**Affiliations:** 1 Department of Endocrinology and Diabetes, Royal Perth Hospital, Perth, WA 6009 Australia; 2 Medical School, University of Western Australia, Nedlands, WA Australia; 3 Department of Endocrinology and Diabetes, Sir Charles Gairdner Hospital, Nedlands, WA Australia; 4 School of Biomedical Sciences, University of Western Australia, Perth, WA Australia; 5 Department of Internal Medicine, Erasmus MC, University Medical Center Rotterdam, Rotterdam, The Netherlands; 6 Estonian Genome Center, Institute of Genomics, University of Tartu, Tartu, Estonia; 7 Center for Child, Adolescent and Maternal Health Research, Faculty of Medicine and Health Technology, Tampere University, Tampere, Finland; 8 Center for Life Course Health Research, Faculty of Medicine, University of Oulu, Oulu, Finland; 9 Division of Endocrinology, Diabetes, and Metabolism, Cedars-Sinai Medical Center, Los Angeles, CA USA; 10 Epidemiology Branch, National Institute of Environmental Health Sciences, Research Triangle Park, North Carolina USA; 11 Division of Genetic Medicine, Department of Medicine, Vanderbilt University Medical Center, Nashville, TN USA; 12 Vanderbilt Genetics Institute, Vanderbilt University Medical Center, Nashville, TN USA; 13 Division of Reproductive Endocrinology and Infertility, Department of Obstetrics and Gynaecology, Erasmus MC, University Medical Center, Rotterdam, the Netherlands; 14 Population Health Sciences, University of Leicester, Leicester, UK; 15 Obstetrics & Gynecology, Medicine, and Healthcare Organization & Policy, Schools of Medicine and Public Health, University of Alabama at Birmingham, Birmingham, AL USA; 16 Department of Obstetrics and Gynecology, Tampere University Hospital, Tampere, Finland; 17 Institute for Molecular Medicine Finland, FIMM, hiLIFE, University of Helsinki, Helsinki, Finland; 18 Medical and Clinical Genetics, University of Helsinki and Helsinki University Hospital, Helsinki, Finland; 19 Department of Twin Research and Genetic Epidemiology, King’s College London, London, UK; 20 MRC Epidemiology Unit, Cambridge Biomedical Campus, University of Cambridge School of Clinical Medicine, Cambridge, UK; 21 Keogh Institute for Medical Research, Nedlands, WA Australia

**Keywords:** Polycystic ovary syndrome, PCOS, GWAS, Meta-analysis, Lean, Obese, Body mass index, BMI

## Abstract

**Background:**

Polycystic ovary syndrome (PCOS) is a complex multifactorial disorder with a substantial genetic component. However, the clinical manifestations of PCOS are heterogeneous with notable differences between lean and obese women, implying a different pathophysiology manifesting in differential body mass index (BMI). We performed a meta-analysis of genome-wide association study (GWAS) data from six well-characterised cohorts, using a case–control study design stratified by BMI, aiming to identify genetic variants associated with lean and overweight/obese PCOS subtypes.

**Results:**

The study comprised 254,588 women (5,937 cases and 248,651 controls) from individual studies performed in Australia, Estonia, Finland, the Netherlands and United States of America, and separated according to three BMI stratifications (lean, overweight and obese). Genome-wide association analyses were performed for each stratification within each cohort, with the data for each BMI group meta-analysed using METAL software. Almost half of the total study population (47%, *n* = 119,584) were of lean BMI (≤ 25 kg/m^2^). Two genome-wide significant loci were identified for lean PCOS, led by rs12000707 within *DENND1A* (*P* = 1.55 × 10^–12^) and rs2228260 within *XBP1* (*P* = 3.68 × 10^–8^). One additional locus, *LINC02905*, was highlighted as significantly associated with lean PCOS through gene-based analyses (*P* = 1.76 × 10^–6^). There were no significant loci observed for the overweight or obese sub-strata when analysed separately, however, when these strata were combined, an association signal led by rs569675099 within *DENND1A* reached genome-wide significance (*P* = 3.22 × 10^–9^) and a gene-based association was identified with *ERBB4* (*P* = 1.59 × 10^–6^). Nineteen of 28 signals identified in previous GWAS, were replicated with consistent allelic effect in the lean stratum. There were less replicated signals in the overweight and obese groups, and only 4 SNPs were replicated in each of the three BMI strata.

**Conclusions:**

Genetic variation at the *XBP1, LINC02905* and *ERBB4* loci were associated with PCOS within unique BMI strata, while *DENND1A* demonstrated associations across multiple strata, providing evidence of both distinct and shared genetic features between lean and overweight/obese PCOS-affected women. This study demonstrated that PCOS-affected women with contrasting body weight are not only phenotypically distinct but also show variation in genetic architecture; lean PCOS women typically display elevated gonadotrophin ratios, lower insulin resistance, higher androgen levels, including adrenal androgens, and more favourable lipid profiles. Overall, these findings add to the growing body of evidence supporting a genetic basis for PCOS as well as differences in genetic patterns relevant to PCOS BMI-subtype.

**Supplementary Information:**

The online version contains supplementary material available at 10.1186/s12864-024-09990-w.

## Background

Polycystic ovary syndrome (PCOS) is a common female endocrinopathy, affecting around 5–15% of women, though its aetiology remains to be fully explained [1]. Cardinal features include hyperandrogenism, oligoamenorrhoea, and often obesity and hyperinsulinaemia [1, 2]. Familial inheritance suggests a genetic basis and genome-wide association studies (GWAS) have identified numerous genetic loci significantly associated with this condition [3–7]. However, the relatively modest number of polymorphisms with robust association data identified to date do not appear to entirely explain the disease aetiology [4]. Most affected women are overweight or obese, with only 16–30% in the lean to normal BMI range [8–10]. Indeed, the clinical manifestations of PCOS are notably different between lean and obese women, potentially implying a different pathophysiology associated with differential body mass index (BMI) [11]. It seems possible that a difference in aetiology is attributable to distinct combinations of genotypes. Improved understanding of the genetic architecture of PCOS subtypes may assist in predicting comorbidity risk, facilitating earlier intervention and tailored patient management. Indeed, principal component analysis has demonstrated clusters of risk factors explaining the variance in PCOS – involving women with i) high BMI, insulin resistance, low high-density lipoprotein and low sex hormone binding globulin, ii) hypertension, elevated low-density lipoprotein and hypertriglyceridemia and iii) a lean PCOS phenotype with elevated luteinizing hormone: follicle stimulating hormone ratio and total testosterone [11]. The lean PCOS phenotype therefore appears to be distinct, potentially necessitating different treatment paradigms, particularly with respect to traditional lifestyle and weight loss recommendations.

In this meta-analysis of PCOS case–control GWAS data we aimed to analyse genotype differences in women with the syndrome based on BMI stratification thus providing insight into the hypothesis that lean and overweight/obese PCOS phenotypes are genetically distinct.

## Results

### Characteristics of the cohorts

A total of 254,588 women were included in the meta-analysis, comprising 5,937 PCOS cases and 248,651 controls stratified into BMI subgroups i) BMI ≤ 25 kg/m^2^ (lean), ii) BMI 25 to 30 kg/m^2^ (overweight), and iii) BMI ≥ 30 kg/m^2^ (obese) (Table 1). Almost half of the combined study subjects (47%, *n* = 119,584) were of lean BMI. The majority of the cases and controls were from Estonian or Finnish datasets, with the remainder comprising American, Australian and Dutch Caucasian subjects (Table 1).

**Table 1:** Details of the cohorts included in the BMI stratified PCOS meta-analysis

**Group**	**Lean**^a^		**Overweight**		**Obese**		**Totals**
	**Case**	**Control**	**Case**	**Control**	**Case**	**Control**	
**WA PCOS**	82	1,287	50	796	139	409	2,763
**Estonian Biobank**	2147	59,319	776	31,579	742	22,980	117,543
**FinnGen**	171	47,533	164	38,656	308	31,605	118,437
**Cedars Sinai**	101	138	66	69	192	60	626
**Dutch cohort**^b^	352	4,643	139	2,130	143	912	8,319
**BioVU**	66	3,745	73	1,388	226	1,402	6,900
**Totals**	**2,919**	**116,665**	**1,268**	**74,618**	**1,750**	**57,368**	**254,588**

^a^Lean = BMI ≤ 25 kg/m^2^, Overweight = 25 < BMI < 30 kg/m^2^, Obese = BMI ≥ 30 kg/m^2^

^b^Cases provided by the Rotterdam PCOS Cohort and controls provided by Lifelines Cohort Study

### Meta-analysis

QQ plots for both the SNP and gene-based analyses completed are presented in Supplementary Fig. 1.

### Single-variant based meta-analysis

For the purposes of this study, genetic loci are defined as regions of the genome containing association signals for PCOS. This study identified two genome-wide significant genetic loci (*P* < 5 × 10^–8^) for lean PCOS (*n* = 2,919 cases and 166,655 controls) on chromosome 9 in *DENND1A* (led by rs12000707; *P* = 1.55 × 10^–12^) and on chromosome 22 in *XBP1* (led by rs2228260; *P* = 3.68 × 10^8^) (Supplementary Fig. 2 and Supplementary Fig. 3). There were no genome-wide significant loci identified for the overweight or obese sub-strata when analysed separately. When the overweight and obese groups were combined (i.e., non-lean subjects), one genome-wide significant locus was identified on chromosome 9, also in *DENND1A* (led by rs569675099; *P* = 3.22 × 10^–9^) (Supplementary Fig. 3 and Supplementary Fig. 4). This variant is in moderate linkage disequilibrium (LD) (r^2^ = 0.51) with rs12000707 (*P* = 3.72 × 10^–8^), which was the lead variant in the lean strata meta-analysis. The variant rs569675099 did not meet GW significance in the lean group (*P* = 1.03 × 10^–5^) though has previously been identified as associated with PCOS in women of European and Han Chinese ancestry [3, 6]. Co-localisation analysis [12] of the GWAS meta-analysis results for the *DENND1A* locus in the lean and non-lean groups generated a 95.5% posterior probability of co-localised association signals in the two datasets, indicating the presence of a shared causal variant.

**Fig. 1:**
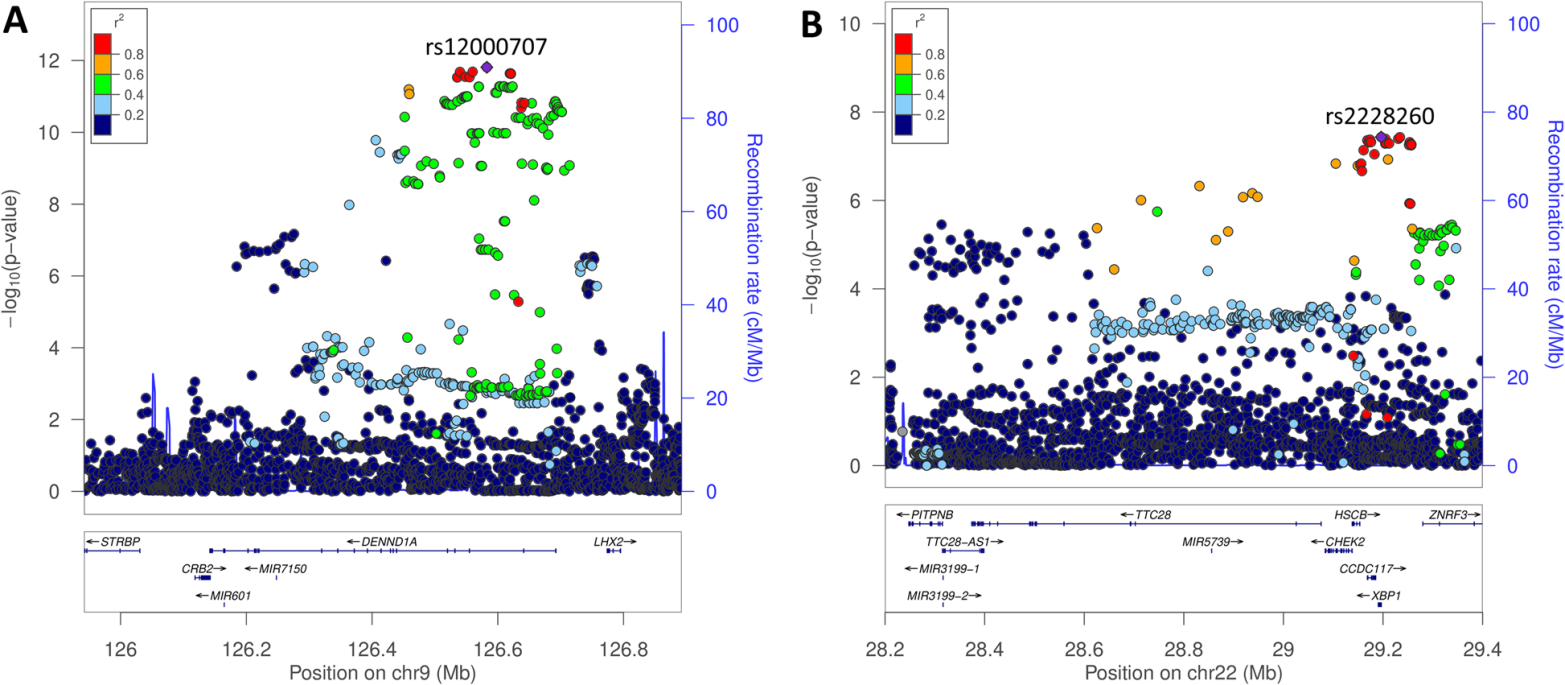
Regional association plots of genome-wide significant loci identified in the lean PCOS meta-analysis. **A** 9q33.3 (*DENND1A*) and **B** 22q12.1 (*XBP1*). Genetic variants are depicted by position (x-axis) together with their meta-analysis *P-*value (-log10; y-axis). Variants are colour coded according to their LD (r^2^) with the lead variant. Mb = million bases

The lead variant in the lean PCOS meta-analysis, rs12000707 (Fig. 1; Supplementary Table 1), is a non-coding intronic variant that has not previously been highlighted by GWAS but does have GTEx data supporting a role as an expression quantitative trait locus (eQTL) in subcutaneous adipose tissue (*DENND1A*; *P* = 7.0 × 10^–6^) [13]. This locus contained a total of 124 genome-wide significant variants in the results for the lean PCOS meta-analysis. The lead single nucleotide polymorphism (SNP), rs12000707 is in complete LD with rs9696009 (r^2^ = 1), previously reported in a GWAS of PCOS conducted in European populations [4]. Data for this locus from FUMA [14] analysis illustrates the large size of the region and number of variants at this site in LD (Supplementary Fig. 5). Based on this data alone it is not possible to determine which variant(s) are the functional drivers within this LD block. The SNP rs12000707 also demonstrated nominally significant associations in the overweight and obese groups (Supplementary Table 1), with meta-analysis of the 3 BMI strata suggesting that there is no significant heterogeneity between the groups (het *P* = 0.56).

The other signal highlighted in the meta-analysis of lean PCOS, on chromosome 22, led by rs2228260, a synonymous SNP in *XBP1* (Fig. 1; Supplementary Table 1), contained a total of 11 genome-wide significant variants Examination of the LD between these variants suggested that they were all likely representative of the same signal (all r^2^ = 1). This association signal is part of a large LD block spanning multiple genes including *TTC28*, *CHEK2*, *HSCB*, *CCDC117*, *XBP1*, *ZNRF3* and *EMID1*. Any one of these genes could be driving the association signal, however publicly available eQTL data from GTEx shows that rs2228260 is an eQTL for *CHEK2* (adrenal gland; *P* = 8.3 × 10^–7^) [13]. This particular variant has not been identified in any previous GWAS. *XBP1* has documented involvement in glucose and lipid metabolism, providing a potential biological link with PCOS, where metabolic disturbances including dyslipidaemia and insulin resistance are noted [11]. However, other genes within this LD block, including both *CHEK2* and *ZNRF3*, have previously been implicated in PCOS by data from the Finnish and Estonian cohorts analysed in isolation, populations included in this meta-analysis [15]. Tyrmi et al., highlighted two putative independent causal variants in the checkpoint kinase 2 (*CHEK2*) gene, which they proposed were the basis of the association [15]. The lead variant for the *CHEK2* locus identified in that study for the Estonian cohort, rs182075939, is not in strong linkage disequilibrium with rs2228260 (r^2^ ≤ 0.2) [16]. Considering this, we performed a conditional analysis for this locus in the lean PCOS meta-analysis using the COJO function of the GCTA package [17]. After conditioning on the *CHEK2* variant rs182075939, the association between rs2228260 and lean PCOS was no longer genome-wide significant (*P*_*cond*_ = 1.9 × 10^–5^), and there was a reduction in effect size (conditioned beta = 0.22, reduced from 0.28). Hence it is not possible to establish rs2228260 as an independent association signal. The lead variant for the Finnish cohort, rs145598156 [15], located closest to *ZNRF3*, and rs2228260 are in linkage equilibrium in Europeans (r^2^ = 0.0) [16]. However, it should be noted that rs145598156 is very rare in non-Finnish Europeans (MAF = 0.003), making accurate estimation of LD difficult. The remaining genes within this LD block have no established biological link with PCOS. The variant rs145598156 was not analysed in this study due to its very low frequency.

The significant associations identified in the lean PCOS meta-analysis were examined within each of the contributing cohorts (Supplementary Table 2). The Estonian Biobank demonstrated the strongest associations of the six cohorts for the two lead variants, which is not surprising considering that this cohort contributed the largest number of lean PCOS cases to the study, while the Western Australian PCOS research group (WA PCOS) cohort demonstrated the greatest effect size for these two variants.

**Fig. 2:**
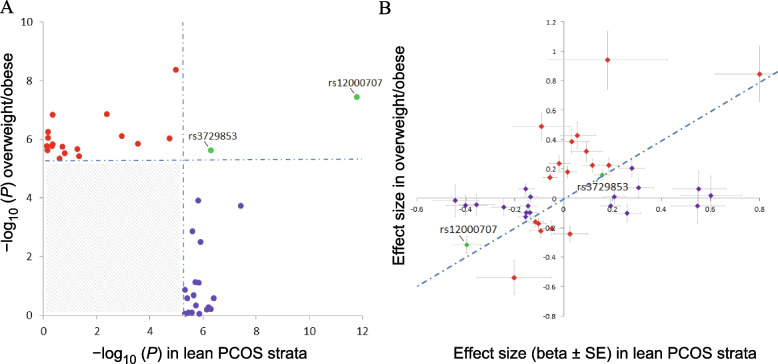
Graph of lead variants in the meta-analyses showing at least suggestive association from each locus. **A** *P*-values are shown from the meta-analyses of the lean PCOS strata (x-axis) or the combined overweight/obese PCOS strata (y-axis). Blue symbols show lead variants with at least suggestive association (*P* < 5 × 10^–6^) in the lean PCOS strata, red symbols show variants with at least suggestive association in the combined overweight/obese PCOS strata and green variants are those with at least suggestive association in both lean PCOS and combined overweight/obese PCOS strata. Lead variants that did not achieve suggestive association fall in the grey region. Dashed lines show the threshold for genome-wide suggestive association (*P* < 5 × 10^–6^). **B** Plot of the effect size (beta ± SE) for the risk allele of lead variants showing at least suggestive association in the lean PCOS strata (blue symbols), combined overweight/obese PCOS strata (red) and variants that show at least suggestive association in both the lean PCOS and combined overweight/obese PCOS strata (green). Dashed line shows the diagonal

Genome-wide suggestive associations may represent true associations that have failed to reach the stringent genome-wide significance threshold for various reasons including statistical power, and which could be validated through further replication. Genetic variants meeting the criterion for genome-wide suggestive association with lean PCOS (*P* < 5 × 10^–6^) are presented in Supplementary Table 1. A number of these signals have previously been identified in GWAS of PCOS affected women of both European and Chinese ancestry, specifically variants in *YAP1*, *KRR1*, *IRF1* and *BLK* [4–7]. However, there has been no previous research published specifically identifying these loci in lean PCOS affected subjects. Furthermore, from the analysis of the combined overweight/obese cohorts, (Supplementary Table 3), genome wide suggestive signals were identified for three previously reported PCOS loci. rs11031006 within *FSHB* was identified as genome-wide suggestive (*P* = 1.42 × 10^–7^), which has been previously reported as a risk variant for PCOS [5, 6, 18]. The other signals in known PCOS loci include rs11453664 on chromosome 2 within *ERBB4* (*P* = 7.85 × 10^–7^) and rs3729853 in *GATA4* on chromosome 8 (*P* = 2.4 × 10^–6^). These specific SNPs have not previously been reported as risk variants for PCOS, though the signal reported in *GATA4*, rs3729853, is in modest LD with the previously published significant SNP rs804279 (r^2^ = 0.36) [4, 16]. Two lead variants were shared by the lean and combined overweight/obese PCOS strata, and with consistent allelic effects observed, when examining results that were of at least suggestive association (*P* < 5 × 10^–6^; Fig. 2). These two variants were located in the *DENND1A* (rs12000707) and *GATA4* (rs3729853) loci. However, the other lead variants that were of at least suggestive association for the lean PCOS stratum and the combined overweight/obese PCOS strata were observed to be uniquely represented in only one stratum or the other (Fig. 2A), suggesting a potential difference in the genetic architecture of lean versus overweight/obese PCOS; the observed effect size of the risk alleles for those lead variants also followed a similar pattern of segregation (Fig. 2B).

### Gene-based association testing

Gene-based association tests are commonly used following single-variant GWAS analysis to model the sum of the effects of all variants within a gene to determine if, despite individual variants not achieving significance, there is statistical evidence of a composite association signal. Gene-based association testing identified two significant associations in the lean PCOS group at the multiple testing corrected (Bonferroni) significance threshold (*P* < 1.96 × 10^–6^) (Supplementary Fig. 6). The leading signal was again *DENND1A* on chromosome 9 (*P* = 4.04 × 10^–10^) followed by *LINC02905* (also known as *C8orf49*) (*P* = 1.76 × 10^–6^), a long intergenic non-protein coding RNA gene located between *GATA4* and *NEIL2* on chromosome 8. The *GATA4*/*NEIL2* locus has been identified as associated with PCOS in previous GWAS in European populations, with heterogeneous effects depending on diagnostic criteria applied [4]. The *GATA4*/*NEIL2* locus also has previous links with ovulatory dysfunction and polycystic ovary morphology [4]. Accordingly, *LINC02905* may be part of a PCOS-susceptibility gene cluster on chromosome 8.

There were no significant gene-based association signals identified for the overweight or obese groups when examined separately, though when combined, one significant signal was identified on chromosome 2 for *ERBB4* (*P* = 1.59 × 10^–6^; Supplementary Fig. 7). This gene has previously been identified in GWAS as associated with PCOS, in women of both European and Chinese ancestry [4, 19, 20].

### Replication of established PCOS loci

All loci associated with PCOS identified in the previously published PCOS GWAS [3–7] were investigated within each of the three BMI strata (Supplementary Table 4). Each locus was examined and observation of *P* < 0.05 in any strata of this meta-analysis was considered nominally significant in terms of replication. For any known loci demonstrating nominal association within the cohort, the beta value or odds ratio was checked for consistent allelic direction of effect with that previously reported. All SNPs known to be associated with PCOS through GWAS were analysed in this study with differing levels of significance across the three BMI strata.

Nineteen of these 28 signals were replicated in the lean cohort, demonstrating consistent allelic effect. There were fewer signals replicated in the overweight and obese groups, with 9 and 7 signals respectively with consistent allelic effect. Only four SNPs, rs9696009 within *DENND1A,* rs2178575 within *ERBB4*, rs11031005 within *ARL14EP/FSHB* and rs1795379 within *KRR1* were replicated in all three BMI strata. The signal within *DENND1A* was most significant in the lean group. The other previously identified SNP in *DENND1A*, rs2479106, did not meet criteria for replication in any BMI tier. The signal within *KRR1* was also most significant in the lean group. The signals in *ERBB4* and *ARL14EP/FSHB* showed similar significance across all BMI strata.

## Discussion

This study aimed to identify differences in genetic architecture between lean, overweight and obese PCOS affected patients in order to provide further insight into potential differences in aetiology between these diverse phenotypes. Single-variant based analysis found evidence of two genome-wide significant genetic associations with the lean phenotype, and one significant association when the overweight and obese groups were combined. Gene-based testing confirmed two genes associated with the lean PCOS group, and one gene associated with the overweight and obese groups combined. Additional variants that demonstrated genome-wide suggestive association were observed in the strata, with lead variants for the lean and combined overweight/obese PCOS strata typically demonstrating greater effects in only one stratum (Figs. 2A, B), therefore, these data may suggest a difference in the genetic architecture underlying lean versus overweight/obese PCOS.

The strongest association signal in the lean analysis, led by rs12000707 on chromosome 9, is located in *DENND1A*, which has robust evidence for genetic involvement in PCOS [4]. This gene encodes the protein DENN/MADD domain containing 1A, which plays a role in endocytosis and receptor turnover and has been identified as associated with PCOS in a number of previous GWAS involving women of European and Han Chinese ancestry [3, 4]. Replication studies have further supported these findings, with certain SNPs identified as associated with increased PCOS risk, highlighting it as one of the most well recognised genes implicated in PCOS [3, 4, 20]. Variants within *DENND1A* have also been associated with hyperandrogenism and ovulatory dysfunction [4, 21]. Functional studies have shown the involvement of *DENND1A* in the pathophysiology of PCOS phenotypes, with laboratory studies demonstrating that ovarian thecal cells in PCOS affected women secrete higher androgen amounts than those from non-affected women, potentially related to upregulation of enzymatic activity in steroid pathways [22]. A DENND1A isoform, termed DENND1A.V2, has been implicated in the increased expression of genes *CYP17A1* and *CYP11A1*, which both play a role in the formation of key enzymes involved in androgen steroidogenesis [23]. This is thought to play a role in PCOS thecal cell androgen production, a feature of PCOS [23]. Furthermore, forced expression of this DENND1A.V2 isoform in normal human theca cells has been shown to increase androgen and progesterone production, thus converting the normal theca cell into a PCOS phenotype [23]. Conversely, a knockdown model whereby DENND1A.V2 expression was silenced in PCOS theca cells demonstrated a reduction in steroidogenesis [23]. A model creating transgenic hDENND1A.V2 mice lines has further demonstrated these concepts; elevation of both ovarian and adrenal Cyp17a1 mRNA levels as well as transgenic ovarian thecal cell steroid production was observed in those mice expressing hDENND1A.V2 transcripts demonstrating impacts on both ovarian and adrenal steroidogenesis [24].

Rare variants within *DENND1A*, identified through whole genome sequencing techniques, have also been found to be associated with certain quantitative traits within PCOS-affected women, specifically higher luteinising hormone (LH): follicle stimulating hormone (FSH) ratios [25]. Clustering analysis, demonstrating ‘reproductive’ and ‘metabolic’ PCOS subgroups, has demonstrated carriers of rare variants in *DENND1A* were more likely to have a reproductive subtype, characterised by lower BMI and insulin levels and higher LH and sex hormone binding globulin levels [26]. The finding of a strong association and large effect size for rs12000707 in the lean PCOS strata with *DENND1A* warrants further investigation. This builds on recent findings of specific *DENND1A* variants being more prevalent within a reproductive PCOS phenotype, with lower BMI [26]. Further studies are necessary to replicate these signals and explore the biology of this gene in PCOS subtypes.

The other genome-wide significant signal in the lean study, led by rs2228260, was located on chromosome 22, within *XBP1.* This signal is composed of a large block of genetic variants in strong LD spanning multiple genes, any one of which could be the effector gene. The SNP identified at this locus is a synonymous coding variant i.e., a codon change that does not alter the encoded amino acid [27]. Synonymous coding variants are not generally regarded as the most likely effectors for transcriptional regulation or altered protein function, but nevertheless, can have effects on protein expression and function. Previously considered ‘silent’ variants, it is now appreciated that these variants can affect mRNA stability and structure, protein folding, conformation and function [28, 29]. Alternatively, this variant may simply be tagging a functional variant that is yet to be identified.

The product of the X-box binding protein 1 (*XBP1*) gene is a transcription factor involved in the ‘Unfolded Protein Response’ (UPR), a series of finely tuned homoeostatic mechanisms triggered by stress within the endoplasmic reticulum (ER) [30]. Dysfunctional ER response has been highlighted as a contributor to the pathogenesis of metabolic disease such as type 2 diabetes, obesity and atherosclerotic cardiovascular disease [30]. Components of the UPR are also known to be involved in the upregulation of metabolic processes, including gluconeogenesis and lipid synthesis, which can be perturbed in PCOS [30]. The protein product of *XBP1* may alter adipocyte, hepatocyte and pancreatic cell signalling pathways to regulate glucose homeostasis and improve insulin sensitivity [30–32]. Deficiency of XBP1 in pancreatic alpha and beta cells has been implicated in impaired insulin secretion and signalling [32]. Increased UPR gene expression has also been seen in granulosa cells in PCOS-affected women and these processes, involving ER stress and associated adaptational mechanisms, have been highlighted as regulators of ovarian physiological and pathophysiological outcomes [33].

XBP1 levels have been reported as higher in women with PCOS [34]. A recent study examining XBP1 levels in three study groups of women: obese PCOS patients, non-obese PCOS patients and normal weight controls, found significantly higher levels in PCOS patients. Comparison between obese and non-obese PCOS affected women found higher levels in the former group and a significant positive correlation was seen between XBP1 levels and BMI, waist circumference, fasting plasma glucose and triglyceride levels [34]. In this context, links with obese PCOS and metabolic characteristics mean that the biological effects of XBP1 in the lean phenotype are not immediately obvious and merit further research. Overall, XBP1 appears to be involved in several processes that are perturbed in PCOS patients including oocyte maturation and aberrant glucose and lipid metabolism. Involvement in the lean phenotype appears to be a novel observation based on the literature to date and warrants further study.

Whilst our top lean PCOS SNP on chromosome 22, rs2228260, is located within *XBP1*, other genes in the region may also be of relevance to this association signal. *CHEK2,* or *Checkpoint Kinase 2,* is one of 7 other genes at this locus harbouring variants in strong LD with rs2228260, and has been associated with PCOS in Finnish and Estonian cohorts [15]. Indeed, rs2228260 has been reported as an eQTL for *CHEK2* in adrenal gland tissue [13]. Furthermore, existing research reporting an association between this gene and PCOS found that it remained significant in the Finnish population after including BMI as a covariate [15]. Given the notable proportion of subjects from these cohorts included in this meta-analysis, it is perhaps unsurprising that this signal is present. It should be noted however that the lead SNP identified in this study differs from that reported in the previous Finnish/Estonian research (rs182075939), the SNPs are not in particularly strong LD (r^2^ < 0.2) [16] and conditioning on rs182075939 did not completely remove the association for the lead SNP identified in this study (although it was no longer genome-wide significant). A recent GWAS found several variants within *CHEK2* to be associated with age at natural menopause (ANM) [35]. There is some evidence of LD between rs5762852 found in that study and our lead PCOS SNP rs2228260 (r^2^ < 0.2, D’ = 1), potentially suggesting shared biology between PCOS and ANM. *CHEK2* is involved in a number of reproductive physiological processes concerning oocyte numbers, follicle atresia, later age at menopause and anti-mullerian hormone (AMH) levels, providing plausible biological links to PCOS [35, 36]. It is possible that there is more than one gene in this chromosomal region driving the associations seen in this and previous studies.

Among the genome-wide suggestive lean PCOS loci, *YAP1*, *KRR1*, *IRF1* and *BLK* are of particular interest. The identification of variants meeting criteria for genome-wide suggestive association with lean PCOS within genes that have previous links to PCOS is encouraging and supports the validity of results. These signals have association with PCOS itself as well as traits involved including ovulatory dysfunction and insulin signalling [4, 37]. Polycystic ovary morphology is also associated with some of these signals, specifically with *YAP1* [4]. Interestingly, the two variants in *YAP1* previously identified as associated with PCOS, rs1894116 and rs11225154 [4, 7] both demonstrated genome-wide suggestive associations in the lean PCOS group but did not reach even nominal significance in the overweight or obese groups. This could suggest that the associations previously reported between this locus and PCOS may be driven primarily by lean PCOS patients. YAP1 (Yes-associated protein 1) has been linked to PCOS pathogenesis through its role in maintaining normal ovarian function, response to gonadotrophins and susceptibility to the effects of androgens [38]. It is involved in a signalling cascade necessary for ovarian function and ovulation, and previous research has supported a *YAP1* mediated mechanism for cell survival and differentiation of granulosa cells during ovulation [39]. This may suggest that this gene contributes to oligoamenorrhoea, and resultant infertility seen in PCOS.

There were 18 SNPs meeting genome wide suggestive association with the combined overweight and obese PCOS strata. Among these, the *TEX41* (testis expressed 41) gene is a non-coding RNA gene, and has been identified as a locus associated with circulating AMH levels in women [40]. The SNP found to be associated with AMH levels, rs13009019, is in strong LD in Europeans (r^2^ = 0.81) with the SNP identified in this study, rs813684 [16], and is thus likely representative of the same signal. On chromosome 22, rs9613552 is found within the gene *TTC28-AS1* (TTC28-Antisense RNA 1). This long non-coding RNA gene has been shown to be downregulated in type 2 diabetes and decreased expression is potentially related to higher risk of developing type 2 diabetes [41]. *CDH18* (cadherin 18 type 2) is one of the closest genes to rs77388455 on chromosome 5. This gene has been reported as associated with phenotypic characteristics common to metabolic syndrome and therefore PCOS, including insulin resistance, glucose intolerance, type 2 diabetes mellitus (T2DM) and obesity [42]**.** The top SNP for this gene had a much lower *P*-value and greater effect size in the non-lean cohort relative to the lean group (*P* = 9.01 × 10^–7^ vs. *P* = 0.64, and beta 0.39 vs 0.03, respectively). Given the increased propensity for PCOS affected women to develop T2DM, and the associated metabolic syndrome type clinical features, it is possible that *CDH18* has some link to PCOS, particularly overweight/obese PCOS. Although signals meeting GW significance were also identified in the overweight/obese groups combined, including *DENND1A*, the strength of association was lower than that seen in the lean cohort. This may imply that PCOS in overweight/obese women is influenced by environment as well as by genetics. Weight gain and high BMI are associated with PCOS-like features such as insulin resistance and oligoamenorrhoea [43, 44]. It is possible that in a proportion of overweight/obese women the strength of the association with genetics is diluted by environmental factors. The relationship between obesity and genetics also needs to be considered, whereby obesity may be regarded as an environmental modifier of PCOS, affecting the emergence of an underlying genetic predisposition as body weight increases.

The gene-based analysis in this study found *LINC02905* to be significantly associated with lean PCOS, in addition to *DENND1A. LINC02905* is a small uncharacterised gene located in between *GATA4* and *NEIL2* in a well-established PCOS susceptibility locus. *LINC02905* is also known as *GATA4* downstream membrane gene (*G4DM*) and is considered to be one of the target genes of *GATA4* [45]. *GATA4* (Gata Binding Protein 4) encodes a member of the GATA family of zinc-finger transcription factors, which is thought to play a role in embryogenesis, myocardial differentiation and function and normal testicular development [46]. Alterations in the expression of *GATA4* have been associated with different types of cancer, including ovarian cancer [45, 46]. Interestingly, this locus has shown heterogeneity of effect in previous research when analyses were compared according to PCOS subtypes, based on different diagnostic criteria. This signal showed stronger association with PCOS defined by NIH criteria (i.e., hyperandrogenism and oligoamenorrhoea) [4].

The other gene highlighted in gene-based analyses was *ERBB4* (v-erb-a erythroblastic leukemia viral oncogene), which was found to be associated with PCOS in the combined overweight/obese group. This gene has previously been associated with PCOS in both women of European ancestry and Han Chinese women [4, 20]. *ERBB4* is a member of the tyrosine protein kinase family and epidermal growth factor receptor subfamily. This gene has been associated with both ovulatory dysfunction and polycystic ovarian morphology, and is hypothesised to be involved in oligoamenorrhoea and infertility aspects of this condition [4, 20]. Furthermore, a murine model, involving *Erbb4* deletion has demonstrated the emergence of various characteristics seen in PCOS patients, specifically disrupted ovulatory cycles with oligomenorrhoea, obesity and impaired oocyte development. In addition, hormonal disturbances included increases in LH and AMH levels, as well as hyperandrogenism [47]. These findings suggest that *ERBB4* may play a key role in PCOS pathophysiology and this is supported by a demonstrated functional role for this gene in ovarian homeostasis and folliculogenesis [47]. The association of this locus specifically with overweight/obese PCOS subjects is a novel finding.

All SNPs previously associated with PCOS were found to be nominally associated with PCOS in at least one of the three BMI stratifications included in this study. A higher proportion of SNPs met criteria for replication in the lean group compared to the individual or combined overweight and obese strata. This is unlikely to be purely due to sample size as the lean and non-lean (combined overweight/obese) groups contained comparable numbers. Some of the SNPs replicated within the lean group have been associated with ovulatory dysfunction and hyperandrogenism, supporting the concept that the lean phenotype is typified by hormonal disturbance and ovarian abnormality, as opposed to the overweight/obese phenotype, which may display a predominance of metabolic disturbance, such as insulin resistance. For example, rs2349415 within *FSHR* has been reported as associated with higher FSH levels as well as ovulatory dysfunction [7, 48]. This SNP demonstrated strongest association with PCOS in the lean group, followed by the overweight group and no association in the obese cohort (*P* = 3.99 × 10^–3^, 0.02 and 0.96, respectively). Similarly, SNPs within *YAP1, TOX3* and *IRF1/RAD50 *were only significant in the lean group, and have previous association with ovulatory dysfunction, hyperandrogenism and increased testosterone levels respectively [4, 7].

The population used for this study comprised a higher proportion of lean women than is described epidemiologically. PCOS-affected women are mostly overweight or obese, with 16–30% falling into a normal or lean BMI category, though around half of cases in this study were BMI < 25 kg/m^2^. Examination of the different cohorts included in the meta-analysis demonstrated varying proportions of women in the BMI strata. The Western Australian PCOS research group aimed to recruit lean women to the study wherever possible to maximise sample size for this group in genetic research, though the proportion of lean/normal BMI women included was just under one third of the WA cohort. The Estonian and Rotterdam cohorts comprised higher proportions of lean women, with 64% and 55% lean cases included respectively. It is noted that these proportions are different to epidemiological reports, though may, in part, be reflective of obesity epidemiology differences between the various cohorts included. Estonia and The Netherlands are known to have lower rates of obesity than Australia and the United States, with data from the US indicating over one third of women in general are obese, compared to 20% in the Netherlands [49]. Overall, a higher number of lean women included in the study improves power for analysis. The previous GWAS available in the literature were conducted in women with BMI within the overweight to obese range, based on WHO definitions, hence this study is different to those previously reported and needs to be considered when drawing comparison. The two large studies in Han Chinese women reported mean BMI within the overweight range for Asian populations, ranging 23.28–24.76 between the two studies and discovery and replication cohorts [3, 7]. The studies conducted in women of European/Caucasian ethnicity included women classified as overweight or obese, with all cases reported to be BMI > 25 [4–6].

## Conclusion

The results from this study provide further evidence to support the theory of genetic differences between lean and overweight/obese PCOS-affected women. Whilst the exact mechanisms by which these signals are contributing to the pathophysiology of this condition are yet to be elucidated, the locations and proximity to a number of genes previously linked with features of PCOS, including ovulatory dysfunction and aberrant metabolism, intimates their potential involvement. Many of the variants identified in this study were intronic, suggesting that they are exerting an effect through modification or enhancement of transcriptional regulation of genes in close proximity (i.e., most often within 200 kb or less) [50], thus influencing the differences in expression of phenotype in these subjects. The findings reported in this study are unique and add to the growing body of evidence supporting both a genetic basis for PCOS as well as differences in genetic patterns based on PCOS phenotype.

## Methods

In this study, a meta-analysis of case–control GWAS data stratified according to BMI was performed. Study subjects were allocated into three groups according to BMI, based on WHO definitions. Lean PCOS was defined by BMI ≤ 25 kg/m^2^, overweight PCOS was defined by BMI > 25—< 30 kg/m^2^ and obese PCOS was defined by BMI ≥ 30 kg/m^2^; control subjects were similarly stratified.

The study subjects used for this analysis were sourced from six separate international cohorts, from the United States of America, Australia, Estonia, Finland and the Netherlands. Each centre recruited PCOS affected women and control subjects of European ancestry or identified them from an existing biobank. For the purposes of study inclusion, PCOS cases were defined according to NIH or Rotterdam criteria, or based on ICD codes and questionnaires (“PCOS coded/self-reported”) depending on the criteria stipulated by each individual centre. Controls were defined as women who did not have a PCOS diagnosis, recruited from population-based samples. Research contributions from the United States of America included the Cedars Sinai (*n* = 359 cases and *n* = 276 controls) [51] and BioVU (*n* = 365 cases and *n* = 6535 controls) [20] cohorts. The Australian cohort was from WA-PCOS (*n* = 271 cases and *n* = 2492 controls) [18, 52]. The contribution from Estonia was from Estonian Biobank (*n* = 3665 cases and *n* = 113,878 controls) [15]. The cohort from Finland was FinnGen (*n* = 643 cases and *n* = 117,794 controls) [15] and from the Netherlands was the Rotterdam PCOS Cohort, with PCOS cases diagnosed in Erasmus Medical Centre, Rotterdam by thorough standardized screening [4] and controls provided by the Lifelines Cohort Study (*n* = 634 cases and *n* = 7685 controls) [53] All these participating cohorts have been described in detail previously.

### Genotyping, quality control and imputation

Cohort-specific information is summarised in Supplementary Table 5. Only individuals from European ancestry were included in the meta-analysis, with each cohort performing adjustment for principal components to correct for any population stratification. GWAS was performed for each cohort using either the SAIGE [54] or SNPTEST [55] software packages. SAIGE software accounts for imbalance in case control ratios, and uses a random effect model. Summary results were supplied from each cohort for meta-analysis, with quality control of the supplied results files performed using EasyQC [56].

### Meta-analysis

Meta-analysis was performed using the METAL software [57]. METAL effectively handles analyses where studies contain disproportionate numbers of cases and controls, thus allowing flexibility, and performs tests for heterogeneity to ensure participating studies demonstrate consistent effects [57]. Meta-analysis was performed using the METAL software using a fixed effects model weighted by standard error [57]. METAL effectively handles analyses where studies contain disproportionate numbers of cases and controls, thus allowing flexibility, and performs tests for heterogeneity to ensure participating studies demonstrate consistent effects [57].

### Annotation and bioinformatics analysis of meta-analysis results

Meta-analysis results were annotated using FUMA software [14]. This platform performs functional mapping and annotation of GWAS results to facilitate interpretation and provide biological context, thus helping to identify causal variants. Both single-variant based annotation and gene-based testing approaches were employed. The FUMA module, SNP2GENE approach, uses submitted GWAS summary statistics to identify lead SNPs and perform functional annotation of all variants in the surrounding genomic regions. Three mapping processes, specifically positional, eQTL and chromatin mapping, work in concert to create a mapped genes table, which in turn is used for the next major function of the FUMA software suite, GENE2FUNC. This process annotates the biological context of these genes thus providing insight into the potential mechanisms of the involved loci [14]. Conditional analysis of the lean PCOS meta-analysis results was performed using the COJO function of the GCTA package [17], which uses GWAS summary statistics and estimated LD from a sample population to identify independent association signals within a genetic locus [58]. The sample genotypes used for LD estimation in the conditional analysis were from the Western Australia cohort. Replication analysis of previously identified PCOS loci was performed for each BMI stratum, with *P* < 0.05 considered nominally significant evidence of replication. Beta values/odds ratios were then examined to confirm a consistent allelic effect to that previously reported. Analysis of the linkage disequilibrium (LD) in regions of interest was performed using LDlink (1000 Genomes Project Phase 3 EUR population) [16]. Expression quantitative trait locus (eQTL) associations were assessed using the GTEx dataset [13]. Co-localisation analysis of GWAS results was performed using the coloc package in R [12], which uses a Bayesian framework to calculate posterior probabilities for 5 different scenarios regarding the presence and co-localisation of association signals in two datasets.

### Supplementary Information

**Additional file 1: Supplementary Figure 1A**. QQ plots for SNP-based analyses for each BMI strata in the meta-analysis of genome-wide association studies for PCOS. A) Lean BMI ≤ 25 kg/m^2^ (λ=1.01) B) overweight 25 < BMI < 30 kg/m^2^ (λ=1.01) C) obese BMI ≥ 30 kg/m^2^ (λ=1.02) D) combined overweight/obese (non-lean) groups (λ=1.01) E) all groups combined (λ=1.04). The lean group demonstrates a greater number of highly significant *p*-values than the overweight and obese groups, likely due to the comparatively larger sample size. **Supplementary Figure 1B**. QQ plots for gene-based analyses for each BMI strata in the meta-analysis of genome-wide association studies for PCOS A) Lean BMI ≤ 25 kg/m^2^ gene-based analysis, B) overweight 25 < BMI < 30 kg/m^2^ gene-based analysis, C) obese BMI ≥ 30 kg/m^2^ gene-based analysis D) combined overweight/obese (non-lean) groups (gene-based analysis), E) all groups combined gene-based analysis.

**Additional file 2: Supplementary Figure 2**. Manhattan plot displaying the results of the lean PCOS single-variant based meta-analysis. Genome-wide significant loci labelled and the threshold for genome wide significance (*P *< 5 x 10^8^) is shown in red.

**Additional file 3: Supplementary Figure 3**. Miami plot depicting the meta-analysis results for the lean (upper panel) and combined overweight/obese PCOS (lower panel) strata. Genome-wide significant loci are labelled and the thresholds for genome-wide significance (*P* < 5 x 10^8^) and genome-wide suggestive significance (*P* < 5 x 10^6^) are shown in red and orange respectively.

**Additional file 4: Supplementary Figure 4**. Manhattan plot displaying the results of the combined overweight/obese PCOS single-variant based meta-analysis. The genome-wide significant locus is labelled and the threshold for genome wide significance (*P *< 5 x 10^8^) is shown in red.

**Additional file 5: Supplementary Figure 5**. Annotation of genome-wide significant signals from meta-analysis of the lean PCOS strata using FUMA software [10]. Plots show the characteristics of each genome-wide significant locus in terms of the physical size, number of potentially relevant SNPs and genes at each locus.

**Additional file 6: Supplementary Figure 6**. Manhattan plot displaying the results from the lean PCOS gene-based meta-analysis. The genome-wide significant genes are labelled and the threshold for genome wide significance (*P *< 1.96 x 10^6^) is shown in red.

**Additional file 7: Supplementary Figure 7**. Manhattan plot displaying the results from the combined overweight/obese PCOS gene-based meta-analysis with a single genome-wide significant gene labelled. The threshold for genome wide significance (*P *< 1.96 x 10^6^) is shown in red.

**Additional file 8: Supplementary Table 1**. Results in each BMI subgroup for loci demonstrating genome-wide suggestive association (*P *<5x10^-6^) with lean PCOS in the individual-variant meta-analysis.

**Additional file 9: Supplementary Table 2**. Cohort-specific results for genome-wide significant association signals identified in the lean PCOS meta-analysis.

**Additional file 10: Supplementary Table 3**. Loci meeting genome-wide suggestive significance (*P* < 5 x 10^-6^) in the combined overweight/obese strata individual-variant based meta-analysis, with comparative data from the lean stratum.

**Additional file 11: Supplementary Table 4**. Summary of loci associated with PCOS from previous GWAS reported in the literature showing the level of significance in this BMI-stratified PCOS meta-analysis.

**Additional file 12: Supplementary Table 5**. Descriptive information for the six cohorts included in the meta-analyses.

**Additional file 13: Supplementary Table 6**. FinnGen Banner Authors 2023.

## Data Availability

The datasets generated and/or analysed during the current study are not publicly available due to embargo instituted by the International PCOS Consortium but are available from the corresponding author on reasonable request.

## Abbreviations

BMI: Body mass index
GWAS: Genome-wide association study
PCOS: Polycystic ovary syndrome
LH: Luteinising hormone
FSH: Follicle stimulating hormone
SHBG: Sex hormone binding globulin
ER: Endoplasmic reticulum
UPR: Unfolded Protein Response
AMH: Anti-Müllerian hormone
FOA: Foetal oocyte attrition
OD: Ovulatory dysfunction
GDM: Gestational diabetes mellitus
eQTL: Expression quantitative trait locus
