# Supplementary material for: Body mass index stratified meta-analysis of genome-wide association studies of polycystic ovary syndrome in women of European ancestry
Source: BMC Genomics. 2024 Feb 26;25:208. doi: 10.1186/s12864-024-09990-w (PMC10895801; doi:10.1186/s12864-024-09990-w)
Supplement: Supplementary file 1 — Additional file 1: Supplementary Figure 1A. QQ plots for SNP-based analyses for each BMI strata in the meta-analysis of genome-wide association studies for PCOS. A) Lean BMI ≤ 25 kg/m2 (λ=1.01) B) overweight 25 < BMI < 30 kg/m2 (λ=1.01) C) obese BMI ≥ 30 kg/m2 (λ=1.02) D) combined overweight/obese (non-lean) groups (λ=1.01) E) all groups combined (λ=1.04). The lean group demonstrates a greater number of highly significant p-values than the overweight and obese groups, likely due to the comparatively larger sample size. Supplementary Figure 1B. QQ plots for gene-based analyses for each BMI strata in the meta-analysis of genome-wide association studies for PCOS A) Lean BMI ≤ 25 kg/m2 gene-based analysis, B) overweight 25 < BMI < 30 kg/m2 gene-based analysis, C) obese BMI ≥ 30 kg/m2 gene-based analysis D) combined overweight/obese (non-lean) groups (gene-based analysis), E) all groups combined gene-based analysis. [file 12864_2024_9990_MOESM1_ESM.docx]

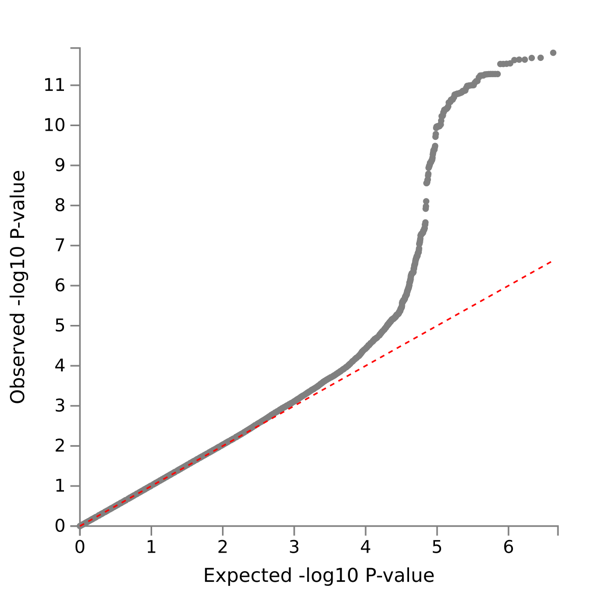

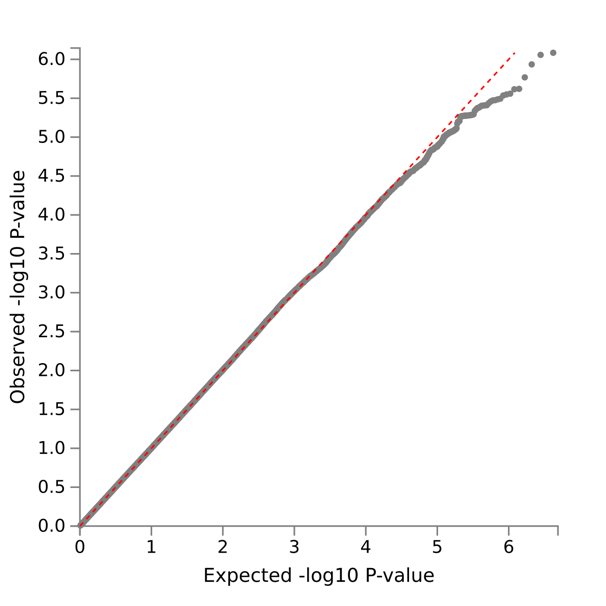


D **Supplementary Figure 1.** QQ plots for each BMI strata in the meta-analysis of genome-wide association studies for PCOS. A) Lean with BMI ≤ 25 kg/m^2^, B) overweight with 25 < BMI < 30 kg/m^2^, C) obese with BMI ≥ 30 kg/m^2^. The lean group demonstrates a greater number of highly significant p-values, likely due to the comparatively larger sample size relative to the two other strata (overweight and obese).

C **Supplementary Figure 1.** QQ plots for each BMI strata in the meta-analysis of genome-wide association studies for PCOS. A) Lean with BMI ≤ 25 kg/m^2^, B) overweight with 25 < BMI < 30 kg/m^2^, C) obese with BMI ≥ 30 kg/m^2^. The lean group demonstrates a greater number of highly significant p-values, likely due to the comparatively larger sample size relative to the two other strata (overweight and obese).

B **Supplementary Figure 1.** QQ plots for each BMI strata in the meta-analysis of genome-wide association studies for PCOS. A) Lean with BMI ≤ 25 kg/m^2^, B) overweight with 25 < BMI < 30 kg/m^2^, C) obese with BMI ≥ 30 kg/m^2^. The lean group demonstrates a greater number of highly significant p-values, likely due to the comparatively larger sample size relative to the two other strata (overweight and obese).

A **Supplementary Figure 1.** QQ plots for each BMI strata in the meta-analysis of genome-wide association studies for PCOS. A) Lean with BMI ≤ 25 kg/m^2^, B) overweight with 25 < BMI < 30 kg/m^2^, C) obese with BMI ≥ 30 kg/m^2^. The lean group demonstrates a greater number of highly significant p-values, likely due to the comparatively larger sample size relative to the two other strata (overweight and obese).


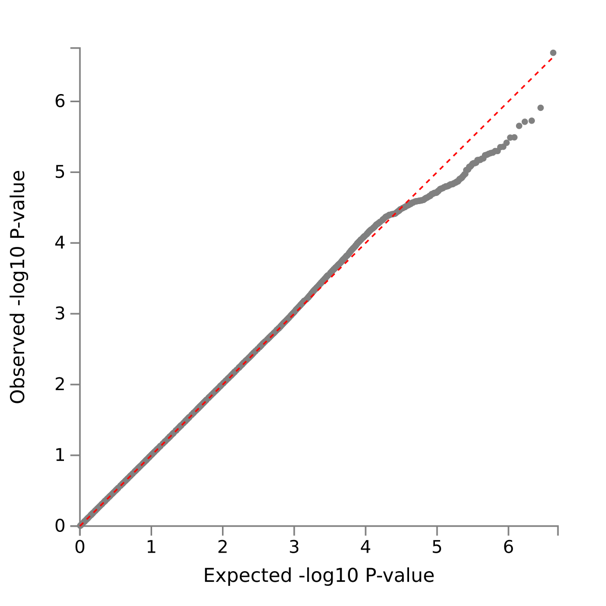

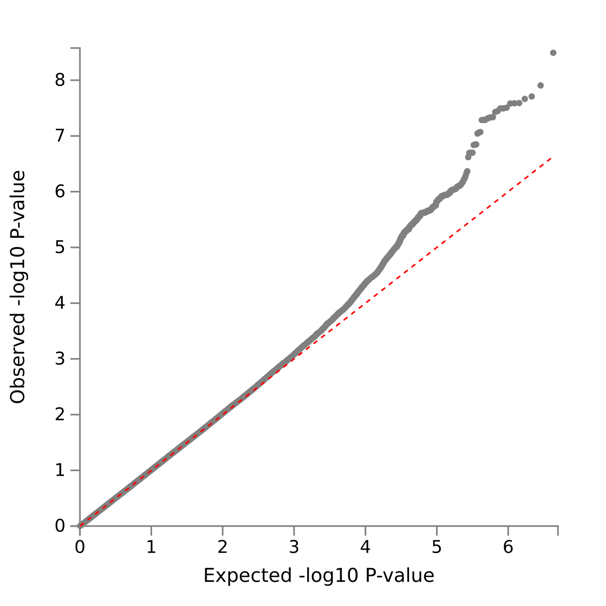


E **Supplementary Figure 1.** QQ plots for each BMI strata in the meta-analysis of genome-wide association studies for PCOS. A) Lean with BMI ≤ 25 kg/m^2^, B) overweight with 25 < BMI < 30 kg/m^2^, C) obese with BMI ≥ 30 kg/m^2^. The lean group demonstrates a greater number of highly significant p-values, likely due to the comparatively larger sample size relative to the two other strata (overweight and obese).


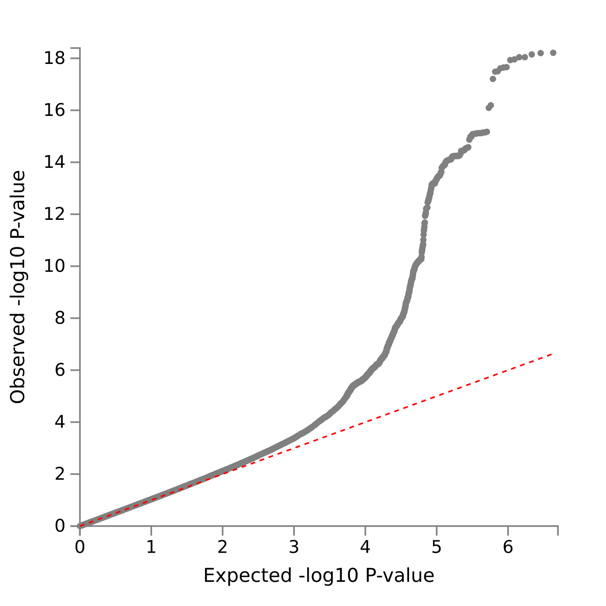


Supplementary Figure 1A. QQ plots for SNP-based analyses for each BMI strata in the meta-analysis of genome-wide association studies for PCOS. A) Lean BMI ≤ 25 kg/m^2^ (λ=1.01) B) overweight 25 < BMI < 30 kg/m^2^ (λ=1.01) C) obese BMI ≥ 30 kg/m^2^ (λ=1.02) D) combined overweight/obese (non-lean) groups (λ=1.01) E) all groups combined (λ=1.04). The lean group demonstrates a greater number of highly significant p-values than the overweight and obese groups, likely due to the comparatively larger sample size.


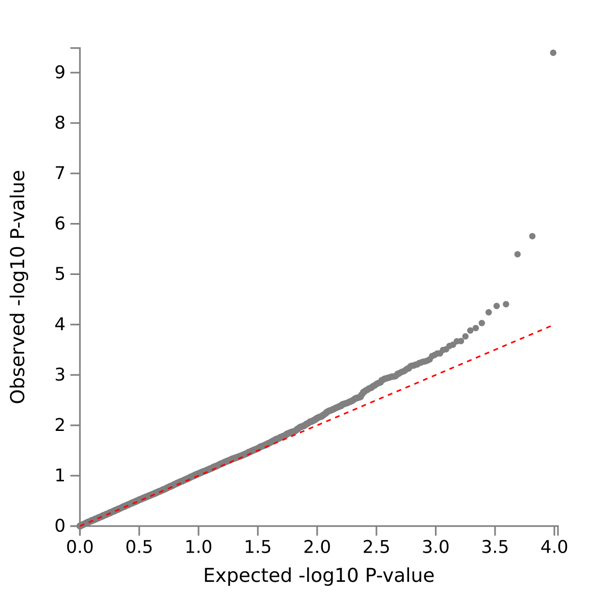

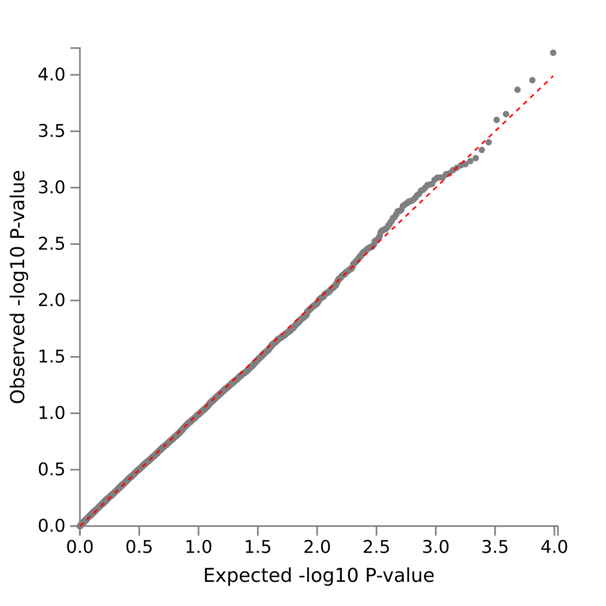


B **Supplementary Figure 1.** QQ plots for each BMI strata in the meta-analysis of genome-wide association studies for PCOS. A) Lean with BMI ≤ 25 kg/m^2^, B) overweight with 25 < BMI < 30 kg/m^2^, C) obese with BMI ≥ 30 kg/m^2^. The lean group demonstrates a greater number of highly significant p-values, likely due to the comparatively larger sample size relative to the two other strata (overweight and obese).

C **Supplementary Figure 1.** QQ plots for each BMI strata in the meta-analysis of genome-wide association studies for PCOS. A) Lean with BMI ≤ 25 kg/m^2^, B) overweight with 25 < BMI < 30 kg/m^2^, C) obese with BMI ≥ 30 kg/m^2^. The lean group demonstrates a greater number of highly significant p-values, likely due to the comparatively larger sample size relative to the two other strata (overweight and obese).

D **Supplementary Figure 1.** QQ plots for each BMI strata in the meta-analysis of genome-wide association studies for PCOS. A) Lean with BMI ≤ 25 kg/m^2^, B) overweight with 25 < BMI < 30 kg/m^2^, C) obese with BMI ≥ 30 kg/m^2^. The lean group demonstrates a greater number of highly significant p-values, likely due to the comparatively larger sample size relative to the two other strata (overweight and obese).

A **Supplementary Figure 1.** QQ plots for each BMI strata in the meta-analysis of genome-wide association studies for PCOS. A) Lean with BMI ≤ 25 kg/m^2^, B) overweight with 25 < BMI < 30 kg/m^2^, C) obese with BMI ≥ 30 kg/m^2^. The lean group demonstrates a greater number of highly significant p-values, likely due to the comparatively larger sample size relative to the two other strata (overweight and obese).


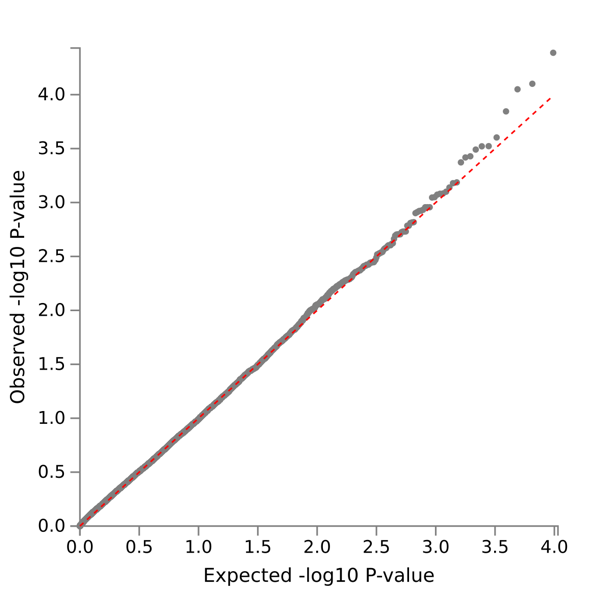

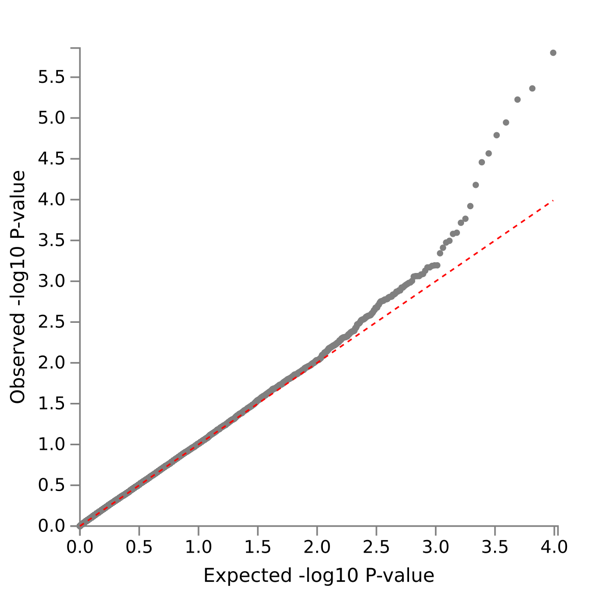

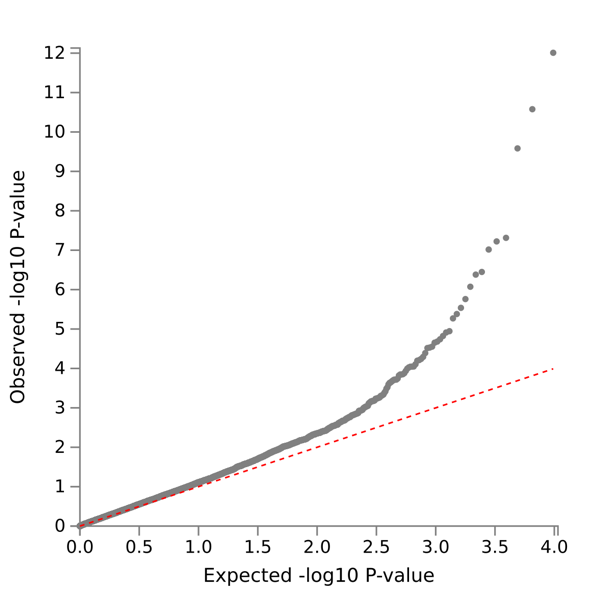


E **Supplementary Figure 1.** QQ plots for each BMI strata in the meta-analysis of genome-wide association studies for PCOS. A) Lean with BMI ≤ 25 kg/m^2^, B) overweight with 25 < BMI < 30 kg/m^2^, C) obese with BMI ≥ 30 kg/m^2^. The lean group demonstrates a greater number of highly significant p-values, likely due to the comparatively larger sample size relative to the two other strata (overweight and obese).

Supplementary Figure 1B. QQ plots for gene-based analyses for each BMI strata in the meta-analysis of genome-wide association studies for PCOS A) Lean BMI ≤ 25 kg/m^2^ gene-based analysis, B) overweight 25 < BMI < 30 kg/m^2^ gene-based analysis, C) obese BMI ≥ 30 kg/m^2^ gene-based analysis D) combined overweight/obese (non-lean) groups (gene-based analysis), E) all groups combined gene-based analysis**.**
