# Supplementary material for: Body mass index stratified meta-analysis of genome-wide association studies of polycystic ovary syndrome in women of European ancestry
Source: BMC Genomics. 2024 Feb 26;25:208. doi: 10.1186/s12864-024-09990-w (PMC10895801; doi:10.1186/s12864-024-09990-w)
Supplement: Supplementary file 2 — Additional file 2: Supplementary Figure 2. Manhattan plot displaying the results of the lean PCOS single-variant based meta-analysis. Genome-wide significant loci labelled and the threshold for genome wide significance (P < 5 x 10^8^) is shown in red. [file 12864_2024_9990_MOESM2_ESM.docx]

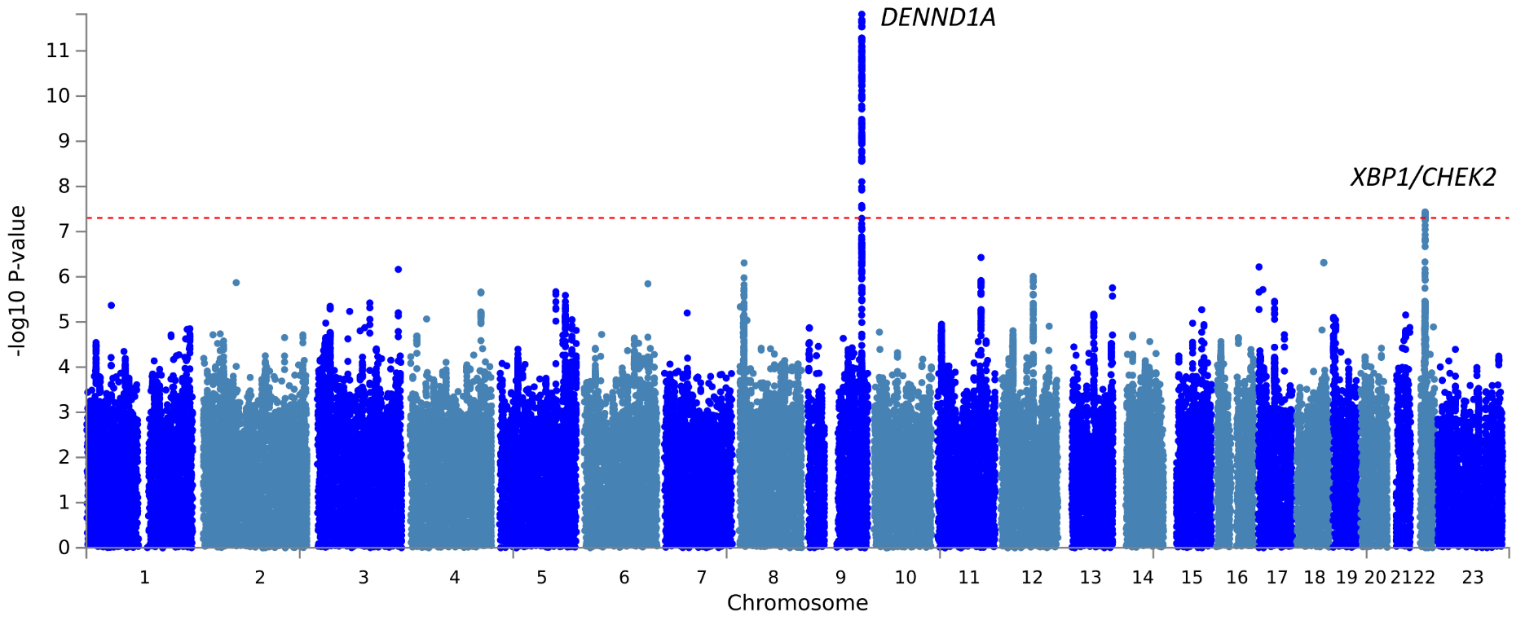


**Supplementary Figure 2**. Manhattan plot displaying the results of the lean PCOS single-variant based meta-analysis. Genome-wide significant loci labelled and the threshold for genome wide significance (*P* < 5 x 10^8^) is shown in red.
