# Supplementary material for: Body mass index stratified meta-analysis of genome-wide association studies of polycystic ovary syndrome in women of European ancestry
Source: BMC Genomics. 2024 Feb 26;25:208. doi: 10.1186/s12864-024-09990-w (PMC10895801; doi:10.1186/s12864-024-09990-w)
Supplement: Supplementary file 3 — Additional file 3: Supplementary Figure 3. Miami plot depicting the meta-analysis results for the lean (upper panel) and combined overweight/obese PCOS (lower panel) strata. Genome-wide significant loci are labelled and the thresholds for genome-wide significance (P < 5 x 10^8^) and genome-wide suggestive significance (P < 5 x 10^6^) are shown in red and orange respectively. [file 12864_2024_9990_MOESM3_ESM.docx]

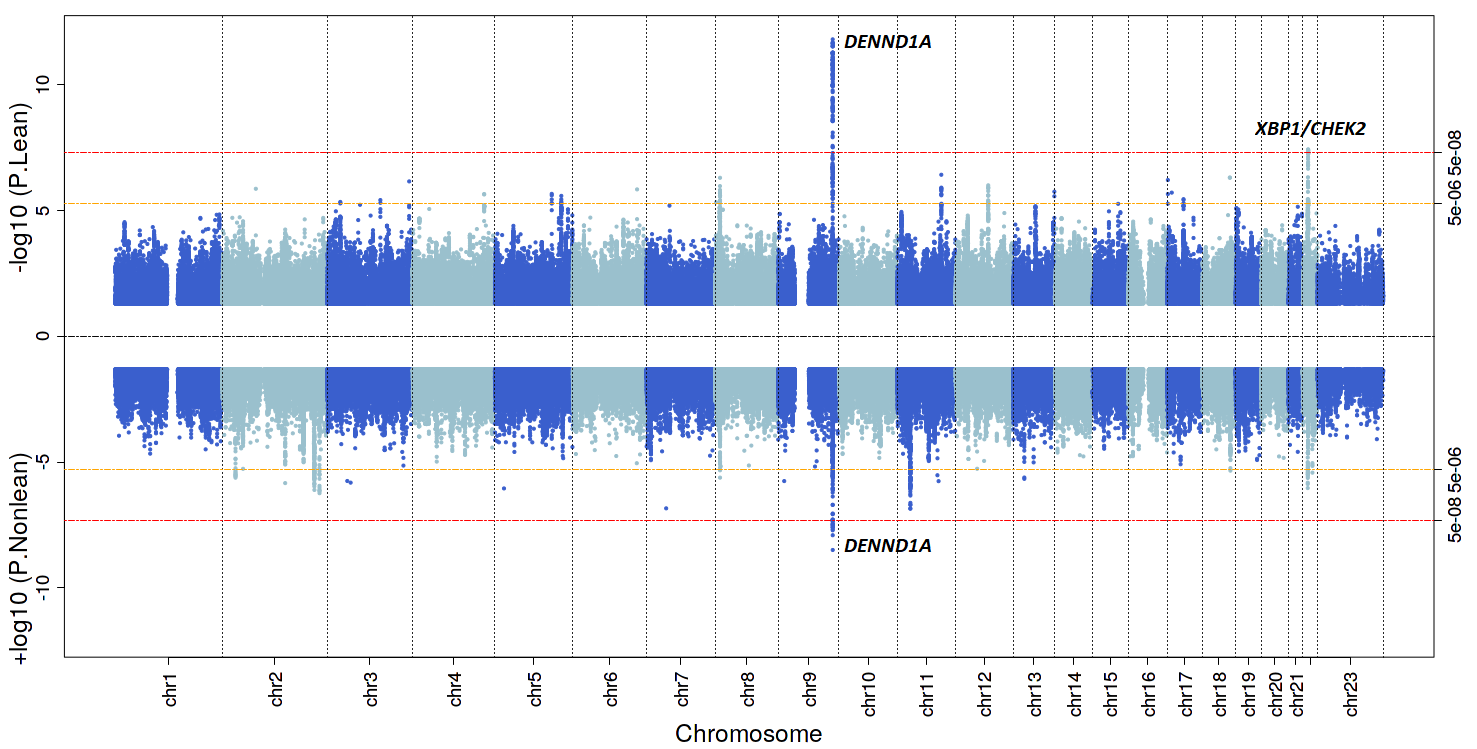


**Supplementary Figure 3.** Miami plot depicting the meta-analysis results for the lean (upper panel) and combined overweight/obese PCOS (lower panel) strata. Genome-wide significant loci are labelled and the thresholds for genome-wide significance (*P* < 5 x 10^8^) and genome-wide suggestive significance (*P* < 5 x 10^6^) are shown in red and orange respectively.
