# Supplementary material for: Body mass index stratified meta-analysis of genome-wide association studies of polycystic ovary syndrome in women of European ancestry
Source: BMC Genomics. 2024 Feb 26;25:208. doi: 10.1186/s12864-024-09990-w (PMC10895801; doi:10.1186/s12864-024-09990-w)
Supplement: Supplementary file 4 — Additional file 4: Supplementary Figure 4. Manhattan plot displaying the results of the combined overweight/obese PCOS single-variant based meta-analysis. The genome-wide significant locus is labelled and the threshold for genome wide significance (P < 5 x 10^8^) is shown in red. [file 12864_2024_9990_MOESM4_ESM.docx]

**
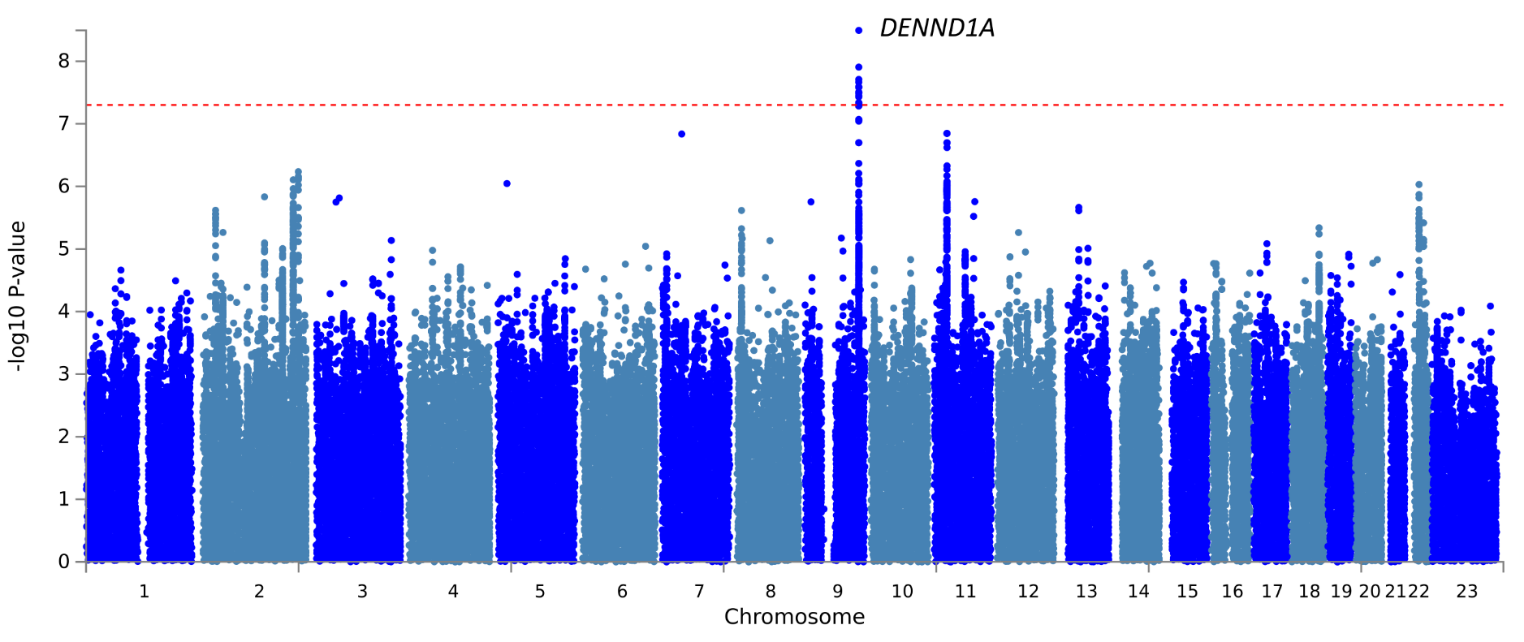
**

**Supplementary Figure 4**. Manhattan plot displaying the results of the combined overweight/obese PCOS single-variant based meta-analysis. The genome-wide significant locus is labelled and the threshold for genome wide significance (*P* < 5 x 10^8^) is shown in red.
