# Supplementary material for: Body mass index stratified meta-analysis of genome-wide association studies of polycystic ovary syndrome in women of European ancestry
Source: BMC Genomics. 2024 Feb 26;25:208. doi: 10.1186/s12864-024-09990-w (PMC10895801; doi:10.1186/s12864-024-09990-w)
Supplement: Supplementary file 5 — Additional file 5: Supplementary Figure 5. Annotation of genome-wide significant signals from meta-analysis of the lean PCOS strata using FUMA software [10]. Plots show the characteristics of each genome-wide significant locus in terms of the physical size, number of potentially relevant SNPs and genes at each locus. [file 12864_2024_9990_MOESM5_ESM.docx]

**
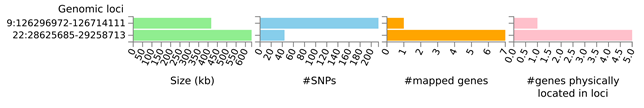
**

**Supplementary Figure 5** Annotation of genome-wide significant signals from meta-analysis of the lean PCOS strata using FUMA software [[10](#_ENREF_10)]. Plots show the characteristics of each genome-wide significant locus in terms of the physical size, number of potentially relevant SNPs and genes at each locus.
