# Supplementary material for: Body mass index stratified meta-analysis of genome-wide association studies of polycystic ovary syndrome in women of European ancestry
Source: BMC Genomics. 2024 Feb 26;25:208. doi: 10.1186/s12864-024-09990-w (PMC10895801; doi:10.1186/s12864-024-09990-w)
Supplement: Supplementary file 6 — Additional file 6: Supplementary Figure 6. Manhattan plot displaying the results from the lean PCOS gene-based meta-analysis. The genome-wide significant genes are labelled and the threshold for genome wide significance (P < 1.96 x 10^6^) is shown in red. [file 12864_2024_9990_MOESM6_ESM.docx]

***~~
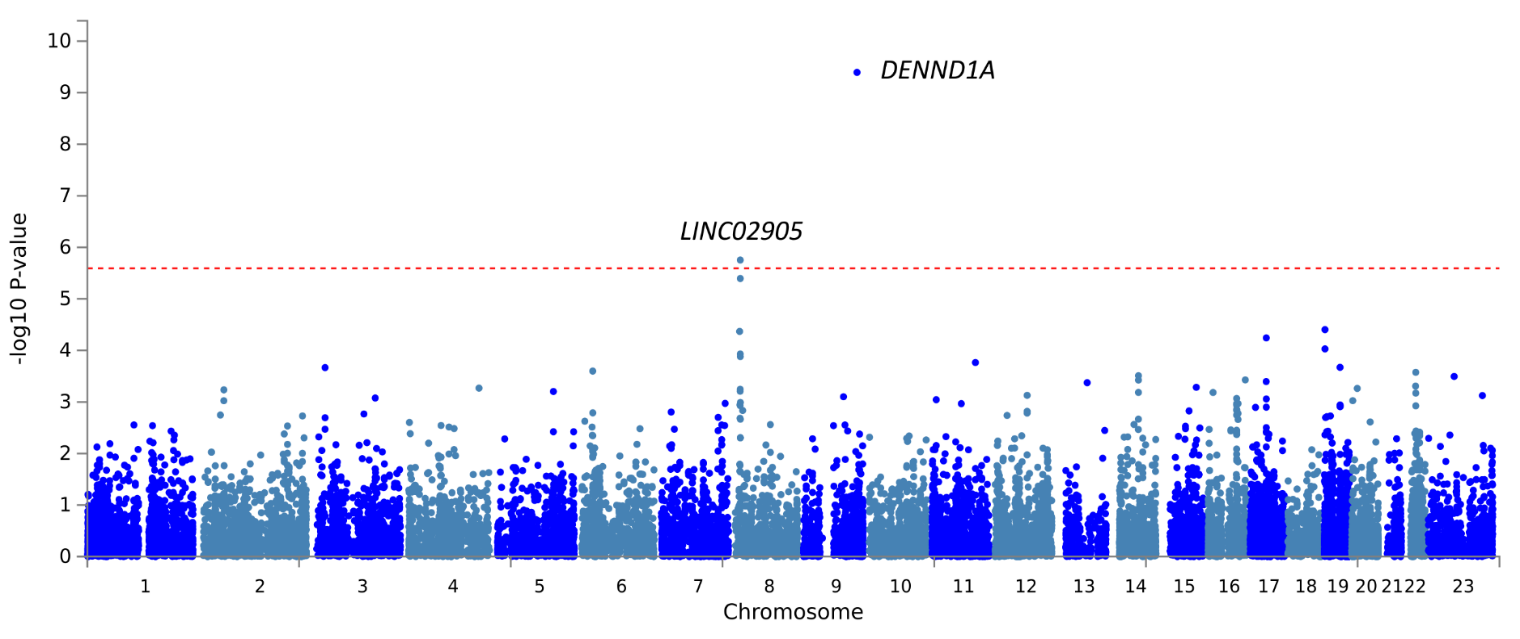
~~***

**Supplementary Figure 6 ~~5~~**. Manhattan plot displaying the results from the lean PCOS gene-based meta-analysis. The genome-wide significant genes are labelled and the threshold for genome wide significance (*P* < 1.96 x 10^6^) is shown in red.
