# Supplementary material for: Body mass index stratified meta-analysis of genome-wide association studies of polycystic ovary syndrome in women of European ancestry
Source: BMC Genomics. 2024 Feb 26;25:208. doi: 10.1186/s12864-024-09990-w (PMC10895801; doi:10.1186/s12864-024-09990-w)
Supplement: Supplementary file 7 — Additional file 7: Supplementary Figure 7. Manhattan plot displaying the results from the combined overweight/obese PCOS gene-based meta-analysis with a single genome-wide significant gene labelled. The threshold for genome wide significance (P < 1.96 x 10^6^) is shown in red. [file 12864_2024_9990_MOESM7_ESM.docx]

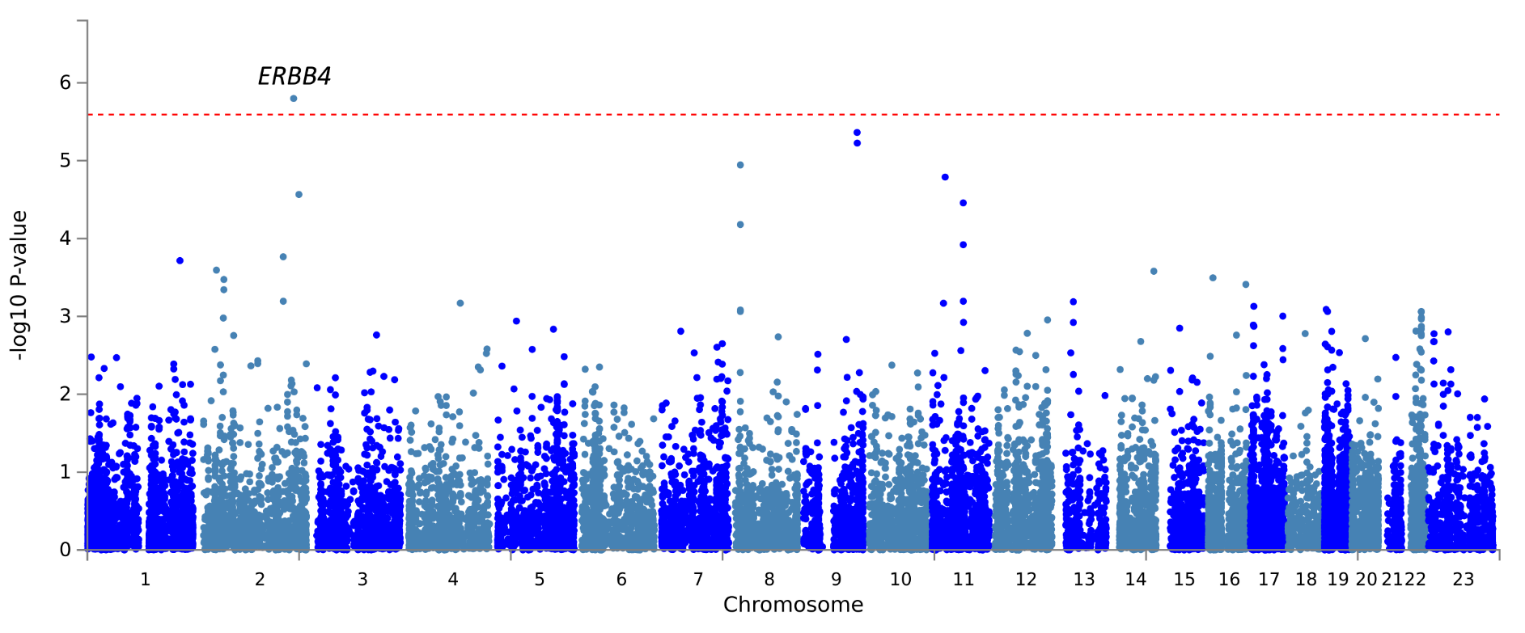


**Supplementary Figure 7 ~~6~~**. Manhattan plot displaying the results from the combined overweight/obese PCOS gene-based meta-analysis with a single genome-wide significant gene labelled. The threshold for genome wide significance (*P* < 1.96 x 10^6^) is shown in red.
