# Supplementary material for: Body mass index stratified meta-analysis of genome-wide association studies of polycystic ovary syndrome in women of European ancestry
Source: BMC Genomics. 2024 Feb 26;25:208. doi: 10.1186/s12864-024-09990-w (PMC10895801; doi:10.1186/s12864-024-09990-w)
Supplement: Supplementary file 8 — Additional file 8: Supplementary Table 1. Results in each BMI subgroup for loci demonstrating genome-wide suggestive association (P <5x10^-6^) with lean PCOS in the individual-variant meta-analysis. [file 12864_2024_9990_MOESM8_ESM.docx]

**Supplementary Table 1.** Results in each BMI subgroup for loci demonstrating genome-wide suggestive association (*P* <5x10^-6^) with lean PCOS in the individual-variant meta-analysis

| **Chr** | **SNP** | **EA** | **OA** | **EAF** | **Nearest Gene** | **Lean** | | **Overweight/Obese** | |
| --- | --- | --- | --- | --- | --- | --- | --- | --- | --- |
|  |  |  |  |  |  | **Effect (OR)** | ***P*-value** | **Effect (OR)** | ***P*-value** |
| 1p32.2 | rs72664661 | A | G | 0.99 | *DAB1* | -2.01 (0.13) | 4.30E-06 | **NA** |  |
| 2p12 | rs71420994 | A | G | 0.02 | *LRRTM4* | 0.60 (1.82) | 1.35E-06 | 0.02 (1.02) | 0.89 |
| 3p24.1 | rs12487145 | A | G | 0.69 | *RBMS3/TGFBR2* | -0.15 (0.86) | 4.51E-06 | -0.05 (0.95) | 0.14 |
| 3q21.1 | rs73188538 | T | C | 0.10 | *HACD2* | -0.24 (0.79) | 3.80E-06 | -0.06 (0.94) | 0.26 |
| 3q28 | rs113705770 | A | C | 0.02 | *CLDN16* | 0.55 (1.73) | 6.88E-07 | -0.05 (0.95) | 0.65 |
| 4q32.3 | rs4691154 | A | G | 0.16 | *TMEM192* | 0.19 (1.21) | 2.18E-06 | -0.05 (0.95) | 0.2125 |
| 5q31.1 | rs2070729 | A | C | 0.47 | *IRF1* | -0.14 (0.87) | 2.47E-06 | -0.10 (0.91) | 1.39E-03 |
| 5q33.2 | rs13190662 | A | G | 0.13 | *LARP1* | 0.21 (1.23) | 2.59E-06 | 0.01 (1.01) | 0.80 |
| 6q25.1 | rs17719900 | A | G | 0.09 | *UST* | 0.26 (1.30) | 1.43E-06 | -0.10 (0.90) | 0.08 |
| 8p23.2 | rs112400145 | T | C | 0.98 | *LOC105377785* | -0.44 (0.64) | 4.63E-06 | -0.02 (0.98) | 0.88 |
| 8p23.1 | rs17807624 | T | C | 0.31 | *BLK* | -0.16 (0.85) | 1.50E-06 | -0.13 (0.88) | 1.26E-04 |
| 9q33.3 | rs12000707* | T | C | 0.93 | *DENND1A* | -0.40 (0.67) | **1.55E-12** | -0.32 (0.73) | **3.72E-08** |
| 11q22.1 | rs111520626 | T | C | 0.06 | *YAP1* | 0.31 (1.36) | 3.74E-07 | 0.07 (1.08) | 0.26 |
| 12q21.2 | rs1148005 | A | G | 0.31 | *KRR1* | -0.15 (0.86) | 1.23E-06 | -0.10 (0.91) | 3.2E-03 |
| 13q34 | rs9550239 | T | C | 0.55 | *TMEM255B* | -0.40 (0.67) | 1.76E-06 | -0.05 (0.95) | 0.47 |
| 17p13.3 | rs77889472 | A | G | 0.95 | *RPA1* | -0.36 (0.70) | 6.06E-07 | -0.04 (0.96) | 0.54 |
| 17p12 | rs112867071 | A | G | 0.66 | *PIRT* | -0.16 (0.85) | 1.92E-06 | 0.06 (1.07) | 0.08 |
| 17q21.1 | rs3902025 | T | G | 0.59 | *GSDMA* | -0.14 (0.87) | 3.50E-06 | 0.01 (1.01) | 0.79 |
| 18q22.1 | rs62095018 | T | C | 0.02 | *CDH19* | 0.55 (1.73) | 4.82E-07 | 0.06 (1.07) | 0.60 |
| 22q12.1 | rs2228260 | A | G | 0.10 | *XBP1* | 0.28 (1.32) | **3.68E-08** | 0.20 (1.22) | 1.92E-04 |

EA: effect allele; OA: other allele; EAF: effect allele frequency; Effect: beta value; OR: Odds ratio. Values in bold are genome-wide significant.* Indicates SNPs with at least GW-suggestive significance in both strata
