# Supplementary material for: Body mass index stratified meta-analysis of genome-wide association studies of polycystic ovary syndrome in women of European ancestry
Source: BMC Genomics. 2024 Feb 26;25:208. doi: 10.1186/s12864-024-09990-w (PMC10895801; doi:10.1186/s12864-024-09990-w)
Supplement: Supplementary file 9 — Additional file 9: Supplementary Table 2. Cohort-specific results for genome-wide significant association signals identified in the lean PCOS meta-analysis. [file 12864_2024_9990_MOESM9_ESM.docx]

**Supplementary Table 2.** Cohort-specific results for genome-wide significant association signals identified in the lean PCOS meta-analysis

|  |  |  | **Western Australia** | | **Estonian Biobank** | | **FinnGen** | | **Cedars Sinai** | | **Rotterdam** | | **BioVU** | | **Meta-Analysis** | | |
| --- | --- | --- | --- | --- | --- | --- | --- | --- | --- | --- | --- | --- | --- | --- | --- | --- | --- |
| **Variant** | **EA** | **EAF** | **Beta** | ***P*** | **Beta** | ***P*** | **Beta** | ***P*** | **Beta** | ***P*** | **Beta** | ***P*** | **Beta** | ***P*** | **Beta (SE)** | ***P*** | **Het *P*** |
| rs12000707 | C | 0.07 | 0.96 | 0.009 | 0.37 | 3.2E-09 | 0.41 | 0.07 | 0.75 | 0.13 | 0.7 | 1.0E-04 | -0.52 | 0.07 | 0.4 (0.06) | **1.6E-12** | 0.02 |
| rs2228260 | A | 0.1 | 1.93 | 0.01 | 0.25 | 3.1E-06 | 0.56 | 0.004 | - | - | 0.54 | 0.21 | - | - | 0.28 (0.05) | **3.7E-08** | 0.06 |

Values in bold are genome-wide significant.
