# Supplementary material for: Body mass index stratified meta-analysis of genome-wide association studies of polycystic ovary syndrome in women of European ancestry
Source: BMC Genomics. 2024 Feb 26;25:208. doi: 10.1186/s12864-024-09990-w (PMC10895801; doi:10.1186/s12864-024-09990-w)
Supplement: Supplementary file 10 — Additional file 10: Supplementary Table 3. Loci meeting genome-wide suggestive significance (P < 5 x 10^-6^) in the combined overweight/obese strata individual-variant based meta-analysis, with comparative data from the lean stratum. [file 12864_2024_9990_MOESM10_ESM.docx]

**Supplementary Table 3.** Loci meeting genome-wide suggestive significance (*P* < 5 x 10^-6^) in the combined overweight/obese strata individual-variant based meta-analysis, with comparative data from the lean stratum.

| **SNP** | **EA** | **OA** | **EAF** | **Nearest Gene** | **Overweight/Obese** | | **Lean** | |
| --- | --- | --- | --- | --- | --- | --- | --- | --- |
|  |  |  |  |  | **Effect (OR)** | ***P*-value** | **Effect (OR)** | ***P*-value** |
| rs11031006 | A | G | 0.14 | *FSHB* | 0.22 (1.25) | 1.42E-07 | 0.12 (1.13) | 3.96E-03 |
| [rs543527054](https://www.ncbi.nlm.nih.gov/snp/rs543527054) | D | I | 0.07 | *DEUP1* | 0.32 (1.38) | 3.01E-06 | 0.09 (1.09) | 1.51E-01 |
| [rs117460293](https://www.ncbi.nlm.nih.gov/snp/rs117460293) | A | G | 0.02 | *MAML2* | 0.94 (2.56) | 1.75E-06 | 0.18 (1.20) | 4.59E-01 |
| [rs9533805](https://www.ncbi.nlm.nih.gov/snp/rs9533805) | A | C | 0.49 | *AX748251* | 0.14 (1.15) | 2.17E-06 | -0.06 (0.94) | 5.06E-02 |
| [rs12965518](https://www.ncbi.nlm.nih.gov/snp/rs12965518) | T | C | 0.87 | *DSEL*/*TMX3* | -0.20 (0.82) | 4.60E-06 | -0.05 (0.95) | 2.46E-01 |
| [rs813684](https://www.ncbi.nlm.nih.gov/snp/rs813684) | T | C | 0.70 | *TEX41* | -0.16 (0.85) | 1.47E-06 | -0.12 (0.89) | 2.81E-04 |
| rs11453664 | D | I | 0.69 | *ERBB4* | -0.17 (0.84) | 7.85E-07 | -0.10 (0.90) | 1.14E-03 |
| [rs12992690](https://www.ncbi.nlm.nih.gov/snp/rs12992690) | T | C | 0.13 | *FAM124B* | 0.24 (1.27) | 5.83E-07 | -0.02 (0.98) | 6.69E-01 |
| [rs906867](https://www.ncbi.nlm.nih.gov/snp/rs906867) | T | C | 0.80 | *LBH* | 0.18 (1.20) | 2.41E-06 | 0.01 (1.01) | 6.79E-01 |
| [rs9613552](https://www.ncbi.nlm.nih.gov/snp/rs9613552) | T | C | 0.13 | *TTC28-AS1* | 0.23 (1.26) | 9.32E-07 | 0.18 (1.20) | 1.75E-05 |
| [rs4821825](https://www.ncbi.nlm.nih.gov/snp/rs4821825) | T | C | 0.88 | *SUN2/DNAL4* | -0.22 (0.80) | 3.83E-06 | -0.09 (0.91) | 4.52E-02 |
| [rs537314545](https://www.ncbi.nlm.nih.gov/snp/rs537314545) | C | G | 0.92 | *LTF/RTP3/LRRC2* | -0.54 (0.58) | 1.78E-06 | -0.20 (0.82) | 1.86E-01 |
| [rs77851804](https://www.ncbi.nlm.nih.gov/snp/rs77851804) | A | G | 0.04 | *CACNA2D3* | 0.43 (1.54) | 1.53E-06 | 0.06 (1.06) | 4.44E-01 |
| [rs77388455](https://www.ncbi.nlm.nih.gov/snp/rs77388455) | A | G | 0.04 | *CDH18/GSUBP1* | 0.39 (1.48) | 9.01E-07 | 0.03 (1.03) | 6.44E-01 |
| [rs56294845](https://www.ncbi.nlm.nih.gov/snp/rs56294845) | T | G | 0.85 | *ADCY1* | 0.49 (1.63) | 1.46E-07 | -0.09 (0.91) | 4.41E-01 |
| [rs3729853](https://www.ncbi.nlm.nih.gov/snp/rs3729853)* | T | C | 0.37 | *GATA4* | 0.16 (1.17) | 2.43E-06 | 0.16 (1.17) | 4.94E-07 |
| rs569675099 | A | G | 0.04 | *DENND1A* | 0.85 (2.34) | **3.22E-09** | 0.80 (2.23) | 1.03E-05 |
| [rs2382453](https://www.ncbi.nlm.nih.gov/snp/rs2382453) | A | G | 0.32 | *NFIB* | -0.24 (0.79) | 1.77E-06 | 0.03 (1.03) | 7.12E-01 |

EA: effect allele; OA: other allele; EAF: effect allele frequency; Effect: beta value; OR: Odds ratio. ID: insertion/deletion variant. Values in bold are genome-wide significant.

* Indicates SNPs with at least GW-suggestive significance in both strata
