# Supplementary material for: Body mass index stratified meta-analysis of genome-wide association studies of polycystic ovary syndrome in women of European ancestry
Source: BMC Genomics. 2024 Feb 26;25:208. doi: 10.1186/s12864-024-09990-w (PMC10895801; doi:10.1186/s12864-024-09990-w)
Supplement: Supplementary file 11 — Additional file 11: Supplementary Table 4. Summary of loci associated with PCOS from previous GWAS reported in the literature showing the level of significance in this BMI-stratified PCOS meta-analysis. [file 12864_2024_9990_MOESM11_ESM.docx]

**Supplementary Table 4.** Summary of loci associated with PCOS from previous GWAS reported in the literature showing the level of significance in this BMI-stratified PCOS meta-analysis.

| **SNP** | **EA** | **EAF** | **Nearest Gene** | **Lean** | | **Overweight** | | **Obese** | |
| --- | --- | --- | --- | --- | --- | --- | --- | --- | --- |
|  |  |  |  | **Beta** | ***P*-value** | **Beta** | ***P*-value** | **Beta** | ***P*-value** |
| rs13405728 | A | 0.89 | *LHCGR* | 0.13 | 0.01 | 0.1 | 0.17 | 0.07 | 0.32 |
| rs2272046 | A | 0.98 | *HMGA2* | 0.22 | 0.03 | 0.19 | 0.21 | 0.23 | 0.1 |
| rs2059807 | A | 0.44 | *INSR* | -0.001 | 0.97 | -0.08 | 0.09 | -0.02 | 0.62 |
| rs6022786 | A | 0.39 | *SUMO1P1* | 0.09 | 2.45E-03 | 0.09 | 0.06 | -0.03 | 0.44 |
| rs12478601 | T | 0.54 | *THADA* | -0.06 | 0.04 | -0.03 | 0.57 | -0.14 | 1.89E-03 |
| rs7563201 | A | 0.5 | *THADA* | -0.11 | 3.06E-04 | -0.08 | 0.07 | -0.18 | 6.59E-05 |
| rs2349415 | T | 0.39 | *FSHR* | 0.09 | 3.99E-03 | 0.11 | 0.02 | -0.002 | 0.96 |
| rs2178575 | A | 0.15 | *ERBB4* | 0.1 | 0.01 | 0.15 | 0.02 | 0.15 | 0.01 |
| rs113168128 | A | 0.01 | *ERBB4* | -0.16 | 0.28 | -0.15 | 0.53 | 0.04 | 0.86 |
| rs4385527 | A | 0.43 | *C9orf3* | -0.09 | 1.21E-03 | -0.14 | 2.31E-03 | -0.05 | 0.26 |
| rs7864171 | A | 0.43 | *C9orf3* | -0.09 | 1.41E-03 | -0.14 | 1.28E-03 | -0.05 | 0.19 |
| rs2479106 | A | 0.59 | *DENND1A* | -0.05 | 0.11 | -0.04 | 0.44 | 0.02 | 0.74 |
| rs9696009 | A | 0.07 | *DENND1A* | 0.39 | 2.30E-12 | 0.33 | 1.14E-04 | 0.28 | 1.59E-04 |
| rs11031005 | T | 0.86 | *ARL14EP/FSHB* | -0.12 | 3.80E-03 | -0.25 | 6.61E-05 | -0.20 | 4.38E-04 |
| rs1894116 | A | 0.91 | *YAP1* | -0.24 | 1.29E-06 | -0.11 | 0.14 | -0.07 | 0.33 |
| rs11225154 | A | 0.09 | *YAP1* | 0.24 | 2.23E-06 | 0.08 | 0.31 | 0.07 | 0.38 |
| rs705702 | A | 0.7 | *ERBB3/RAB5* | -0.08 | 0.02 | -0.06 | 0.19 | -0.11 | 0.01 |
| rs2271194 | T | 0.39 | *ERBB3/RAB5* | 0.06 | 0.04 | 0.112 | 0.01 | 0.08 | 0.07 |
| rs4784165 | T | 0.77 | *TOX3* | -0.08 | 0.01 | -0.09 | 0.09 | -0.08 | 0.1 |
| rs8043701 | A | 0.81 | *TOX3* | -0.13 | 3.09E-04 | -0.07 | 0.2 | -0.07 | 0.2 |
| rs144248326 | C | 0.98 | *WWTR1* | 0.08 | 0.78 | 0.51 | 0.14 | -0.13 | 0.62 |
| rs13164856 | T | 0.69 | *IRF1/RAD50* | 0.11 | 8.59E-04 | 0.06 | 0.21 | 0.06 | 0.19 |
| rs17186366 | C | 0.93 | *SOD2* | -0.01 | 0.89 | -0.04 | 0.63 | -0.1 | 0.23 |
| rs804279 | A | 0.22 | *GATA4/NEIL2* | 0.1 | 4.23E-03 | 0.16 | 2.82E-03 | 0.04 | 0.42 |
| rs10739076 | A | 0.28 | *PLGRKT* | -0.12 | 0.15 | 0.08 | 0.4 | -0.15 | 0.07 |
| rs1784692 | T | 0.87 | *ZBTB16* | 0.16 | 1.93E-04 | 0.15 | 0.02 | 0.15 | 0.05 |
| rs1795379 | T | 0.31 | *KRR1* | -0.14 | 4.35E-06 | -0.11 | 0.03 | -0.09 | 0.04 |
| rs853854 | T | 0.52 | *MAPRE1* | -0.02 | 0.86 | -0.02 | 0.86 | -0.14 | 0.09 |

EA: effect allele; OA: other allele; EAF: effect allele frequency;
