# Supplementary material for: Body mass index stratified meta-analysis of genome-wide association studies of polycystic ovary syndrome in women of European ancestry
Source: BMC Genomics. 2024 Feb 26;25:208. doi: 10.1186/s12864-024-09990-w (PMC10895801; doi:10.1186/s12864-024-09990-w)
Supplement: Supplementary file 12 — Additional file 12: Supplementary Table 5. Descriptive information for the six cohorts included in the meta-analyses. [file 12864_2024_9990_MOESM12_ESM.docx]

**Supplementary Table 5.** Descriptive information for the six cohorts included in the meta-analyses

| **Cohort** | **Diagnostic criteria** | **Genotyping array** | **Pre-imputation QC** | | | | **Imputation panel** | **Imputation software** | **GWAS analysis software** |
| --- | --- | --- | --- | --- | --- | --- | --- | --- | --- |
|  |  |  | **HWE** | **MAF** | **SNP** | **Sample** |  |  |  |
| WA-PCOS | NIH | Illumina OmniExpress, HumanHap610 | >0.01 | ≥1% | ≥95% | ≥95% | 1000G Phase3 | Minimac4 | SAIGE |
| EstBB | ICD codes | Illumina GSAv1.0, GSAv2.0, GSAv2.0_EST | >10^-4^ | ≥1% | ≥95% | ≥95% | Estonian population specific panel[[52](#_ENREF_52)] | Beagle v.28Sep18.793 | SAIGE |
| FinnGen | ICD codes | Affymetrix Axiom FinnGen custom arrays | >10^-6^ | ≥1% | ≥98% | ≥95% | SISu panel v3.0 (Finnish specific) | Beagle 4.1 | SAIGE |
| Cedars | NIH, self-reported | Illumina GSA (v2 and v3) | >10^-6^ | ≥1% | ≥98% | ≥95% | HRC r1.1 | PBWT | SAIGE |
| Dutch cohort* | Rotterdam | Illumina GSA | >0.01 | ≥1% | ≥95% | ≥95% | 1000G Phase3 | IMPUTE2 v2.3.2 | SNPTEST |
| BioVU | ICD codes | Illumina MEGA | >10^-10^ | ≥1% | ≥95% | ≥98% | HRC r1.1 | Minimac4 | SAIGE |

HWE: Hardy-Weinberg Equilibrium *P*-value threshold; MAF: minor allele frequency threshold; SNP: genetic variant call-rate threshold; Sample: sample call-rate threshold.

*****Cases provided by the Rotterdam PCOS Cohort and controls provided by Lifelines Cohort Study
